# Sensitivity and Specificity of Treponemal-specific Tests for the Diagnosis of Syphilis

**DOI:** 10.1093/cid/ciaa349

**Published:** 2020-06-24

**Authors:** Ina U Park, Anthony Tran, Lara Pereira, Yetunde Fakile

**Affiliations:** 1 Division of STD Prevention, National Center for HIV/AIDS, Viral Hepatitis, STD, and TB Prevention, Centers for Disease Control and Prevention, Atlanta, Georgia, USA; 2 Department of Family and Community Medicine, University of California San Francisco School of Medicine, San Francisco, California, USA; 3 Public Health Laboratory, Department of Forensic Sciences, Washington, DC, USA

**Keywords:** syphilis, diagnostics, test performance, treponemal, immunoassay

## Abstract

We conducted a systematic review of relevant syphilis diagnostic literature to address the question, “What is the sensitivity and specificity of the treponemal tests currently approved by the Food and Drug Administration (FDA) for the diagnosis of syphilis (by stage)?” There were 16 treponemal assays evaluated: 13 immunoassays and 3 manual assays (fluorescent treponemal antibody absorbed test [FTA-ABS], microhemagglutination assay for *Treponema pallidum* antibodies [MHA-TP], *Treponema pallidum* particle agglutination assay [TP-PA]). MHA-TP and FTA-ABS were less sensitive in primary and secondary syphilis than TP-PA; TP-PA is the most specific manual treponemal assay. There is insufficient evidence to recommend one particular treponemal immunoassay (eg, enzyme immunoassays, chemiluminescence immunoassays, microbead immunoassays) over another based on published performance data. For diagnosis of neurosyphilis, cerebrospinal fluid (CSF) TP-PA has similar performance to CSF FTA-ABS in studies with patients with definitive or presumptive neurosyphilis. However, CSF treponemal testing has limitations in its sensitivity and specificity and should be interpreted within the context of the clinical scenario, additional CSF test results and syphilis prevalence.

Laboratory diagnosis of syphilis has traditionally involved an algorithm beginning with a nontreponemal test (eg, rapid plasma regain [RPR]) followed by a manual *Treponema pallidum–*specific assay (eg, *T. pallidum* particle agglutination assay [TP-PA]) for confirmation of reactive nontreponemal serology. Currently, various treponemal-specific immunoassays are increasingly being used for syphilis screening and diagnosis, including enzyme immunoassays (EIAs), chemiluminescence immunoassays (CIAs), and microbead immunoassays (MBIAs), among others. These assays can be automated, reducing labor and turnaround time. Because some of these assays are relatively nonspecific, a reverse-sequence algorithm has been employed beginning with a treponemal immunoassay, followed by reflex nontreponemal testing (eg, RPR) on initially reactive specimens [1]. Currently, the Centers for Disease Control and Prevention (CDC) recommends conducting a TP-PA if there are discordant results between the immunoassay and RPR (eg, EIA-reactive, RPR-nonreactive) [1]. Regardless of which algorithm is used, for laboratories to select the most appropriate treponemal test(s) it is important to consider the sensitivity and specificity of these assays in clinically characterized sera, stratified by stage of syphilis.

We conducted a systematic review of the literature on the test performance of treponemal-specific tests, and results of this review were presented to a national consultation of experts in November 2017. Our review was based on a single key question: What is the sensitivity and specificity of the treponemal tests currently approved by the Food and Drug Administration (FDA) for the diagnosis of syphilis (by stage)? Our objective of this review was to inform the selection of the appropriate confirmatory treponemal test for laboratories using the traditional algorithm. These data will assist laboratories in their selection of an initial treponemal test when the reverse sequence algorithm is used for diagnosis of syphilis. Additionally, the data will facilitate selection of the appropriate second treponemal test for patients with initially discordant treponemal and nontreponemal serology (eg, CIA-reactive, RPR-nonreactive).

## METHODS

We searched Medline, Embase, Scopus, Cochrane Library, and CINAHL from 1960 to 30 June 2017. Following the consultation in November 2017, we subsequently updated the literature search from July 2017 to September 2018 using the following search terms: (Treponema pallidum OR Neurosyphilis OR Syphilis) AND (sero-diagnos* OR serodiagnos* OR (serolog* AND (test* OR exam* OR assay* OR screen* OR lab* OR diagnos* OR nontreponemal OR treponemal OR algorithm* OR antibody titer) OR serofast)). The search was limited to human studies published in English.

The initial search yielded n = 4851 nonduplicated abstracts. We excluded n = 4504 abstracts that were not relevant to the key question: studies of nontreponemal testing only, animal studies, direct detection studies, review articles, guidelines, letters to the editor, and other publications that were not primary research studies. We reviewed 347 abstracts, and further excluded n = 230 studies that described obsolete tests only, tests not approved by the FDA, those that used a gold standard based exclusively on non-FDA approved tests, studies of prevalence or laboratory technique only (no test performance), any duplicate publications, and abstracts without a full manuscript. After exclusions, 117 full papers were reviewed for potential inclusion, 81 studies with either descriptive data on use of treponemal tests or actual test performance data were abstracted into Tables of Evidence (Supplementary Table)

Studies with test performance data were prioritized according to their relevance to the key question (Supplementary Table). Studies of high relevance were those with clinically characterized specimens, stratified by stage of syphilis (with/without use of dark-field microscopy for diagnosis of primary syphilis), and included studies that utilized syphilis specimens from commercial or CDC serum banks. Studies of moderate relevance were those with clinically characterized specimens but no stratification by stage (all patients with syphilis analyzed together). Lower relevance studies were those that used a laboratory reference standard only (single or multiple tests) without clinical characterization, and also include studies where clinical characterization could not be assessed or was not performed uniformly across specimens. Studies of high and moderate relevance were abstracted into tables of test performance, and the range of sensitivity and specificity estimates from all studies was abstracted. If only a single study was available for a particular assay, the proportions (n/N) and 95% confidence intervals were abstracted.

Following presentation of the published test performance data at the national consultation, it was noted that many of the treponemal immunoassays had little or no data on test performance published in the peer-reviewed literature. Therefore, for the treponemal immunoassays, we obtained 510(k) Premarket Notification data submitted to the FDA and also abstracted these data into the Tables of Evidence.

## RESULTS

A summary of characteristics of FDA-approved treponemal tests, including manufacturer, assay type, antigens, antibodies detected, and specimen type, is detailed in Table 1. Among the 16 treponemal assays reviewed, there were 13 immunoassays and 3 manual assays: fluorescent treponemal antibody absorbed test (FTA-ABS), microhemagglutination assay for *Treponema pallidum* antibodies (MHA-TP), and TP-PA. Ten treponemal tests had published data on sensitivity and specificity. Two immunoassays had performance data that were not stratified by stage of syphilis (Abbott Architect, Roche Elecsys). Among the other 8 that had data stratified by stage of syphilis, 3 were manual treponemal assays and 5 were immunoassays (ADVIA Centaur, Bioplex 2200, Captia Syphilis, G, LIAISON, Trep-Sure). The performance characteristics for these 10 treponemal assays were summarized from 21 highly relevant studies and 11 moderately relevant studies in Tables 2 and 3.

**Table 1.** Food and Drug Administration–Approved Treponemal-specific Tests

Assay (Manufacturer)	Assay Type	Antigens	Antibodies	Sample Types
Immunoassays				
ADVIA Centaur (Siemens)	CIA	Recombinant TpN15, TpN17	Not specified	Serum, heparinized plasma, EDTA plasma, citrate plasma
Architect Syphilis TP (Abbott)	CMIA	Recombinant TpN15, TpN17, TpN47	IgG, IgM	Serum, plasma
AtheNA Multi-Lyte T. pallidum IgG Plus Test System (Zeus Scientific)	MFIA	Recombinant TpN17	IgG	Serum
Bioplex 2200 Syphilis IgG (Biorad)	MFIA	Recombinant TpN15, TpN17, TpN47	IgG	Serum
Bioplex 2200 Syphilis Total and RPR (Biorad)	MFIA	Recombinant TPN17, TPN47	IgG, IgM	Serum, heparinized plasma, EDTA plasma
Captia Syphilis-G Assay (Trinity Biotech)	EIA	Wild-type antigens	IgG	Serum
Elecsys Syphilis (Roche)	CIA	Recombinant TpN15, TpN17, TpN47	IgG, IgM	Serum, heparinized plasma, EDTA plasma, citrate plasma
Enzy-Well Syphilis IgG (Diesse Diagnostica Senese)	ELISA	Recombinant antigens (proprietary)	IgG	Serum, plasma
Immulite 2000 Syphilis Screen (Siemens)	CIA	Recombinant TpN17	Not specified	Serum, heparinized plasma
LIAISON (Diasorin)	CIA	Recombinant TpN17	IgG, IgM	Serum
Lumipulse G TP-N (Fujirebio)	CIA	Recombinant TpN15, TpN17, TpN47	IgG, IgM	Serum, EDTA plasma, citrate plasma
Trep-Sure (Trinity Biotech)	EIA	Recombinant antigens (proprietary)	IgG, IgM	Serum, plasma
Zeus Scientific T Pallidum IgG Test System (Zeus Scientific)	ELISA	Recombinant TpN17	IgG	Serum
Manual assays				
FTA-ABS	Indirect fluorescence	Wild-type, fixed to slide	IgG, IgM	Serum
MHA-TP	Agglutination	Wild-type, bound to sheep erythrocytes	IgG, IgM	Serum, plasma
TP-PA (Fujireibo)	Agglutination	Wild-type, bound to gelatin particles	IgG, IgM	Serum, plasma

Abbreviations: CIA, chemiluminescence immunoassay; CMIA, chemiluminescent microparticle immunoassay; EDTA, ethylenediaminetetraacetic acid; EIA, enzyme immunoassay; ELISA, enzyme-linked immunosorbent assay; FTA-ABS, fluorescent treponemal antibody absorbed test; Ig, immunoglobulin; MFIA, multiplex flow immunoassay; MHA-TP, microhemagluttination assay for *T. pallidum* antibodies; TP-PA, *Treponema pallidum* particle agglutination assay.

**Table 2.** Sensitivity and Specificity of Manual Treponemal Assays for Diagnosis of Syphilis in Clinically Characterized Specimens (Published Data)

	Test Performance			
Assay	Stage	Sensitivity	Specificity	References
FTA-ABS	Primary	78.2–100%	87.0–100%	Augenbraun [2], Byrne [3], Coffey [4], Farshy [5], Huber [6], Ijsselmuiden [7], Ijsselmuiden [8], Jaffe [9], Larsen [10], Lam [11], Moyer [12], Park [13], Pope [14], Romanowski [15], Van Eijk [16], Young [17]
	Secondary	92.8–100%		
	Latent (combined)	83–100%		
	Early latent	94.4–100%		
	Late latent	84.5–92.6%		
MHA-TP	Primary	45.9–88.6%	98.8–99.0%	Augenbraun [2], Coffey [4], Dyckman [18], Huber [6], Jaffe [9], Larsen [10], Pope [14]
	Secondary	90–100%		
	Latent (combined)	99–100%c`		
	Early latent	94.4–100%		
	Late latent	97%		
TP-PA	Primary	86.2–100%	99.6–100%	Bosshard [19], Creegan [20], Cole [21], Lam [11], Liu [22], Manavi [23], Park [13], Pope [24], Wellinghausen [25]
	Secondary	100%		
	Latent (combined)	100%		
	Early latent	94.4–100%		
	Late latent	86.8–100%		

Abbreviations: FTA-ABS, fluorescent treponemal antibody absorbed test; MHA-TP, microhemagluttination assay for *T. pallidum* antibodies; TP-PA, *Treponema pallidum* particle agglutination assay.

**Table 3.** Sensitivity and Specificity of Treponemal Immunoassays for Diagnosis of Syphilis in Clinically Characterized Specimens (Published Data)

	Test performance, % (95% CI)			
Assay	Stage	Sensitivity	Specificity	References
ADVIA Centaur	Primary	(52/55), 94.5% (84.9–98.9)	(385/403), 95.5% (93.0–97.3)	Park [13]
	Secondary	(98/98), 100% (96.2–100)		
	Early latent	(41/41), 100% (90.7–100)		
	Late latent	(64/68), 94.1% (85.6–98.4)		
	Overall	(255/262) 97.3% (94.6–98.9)		
Architect Syphilis	Overall	97.3–100%	94.5–100%	Liu [22], Marangoni [26], Saral [27], Wellinghausen [25], Xia [28], Xu [29]
Bioplex 2200 Syphilis IgG	Primary	(53/55), 96.4% (94.5–98.2)	(390/403), 96.9% (94.1–98.7)	Park [13]
	Secondary	(98/98), 100% (96.2–100)		
	Early latent	(39/41), 95.1% (83.8–99.4)		
	Late latent	(64/68), 94.1% (85.6–98.4)		
	Overall	(264/262), 96.9% (94.1–98.7)		
Captia Syphilis-G Assay	Primary	82.3–100%	97.8–100%	Cole [21], Lefevre [30], Siletti [31], Young [17, 32, 33]
	Secondary	100%		
	Early latent	100%		
	Late latent	91.7–100%		
	Overall	94.7–100%		
Elecsys Syphilis	Overall	(57/57), 100% (93.9–100)	(519/527), 98.5% (97.0–99.3)	Xia [28]
LIAISON	Primary	96.4–100%	94.5–100%	Marangoni [34], Park [13], Wellinghausen [25]
	Secondary	100%		
	Latent (combined)	96.1%		
	Early latent	97.6%		
	Late latent	92.6%		
	Overall	94.5–100%		
Trep-Sure	Primary	(52/55), 94.5% (84.9–98.9) [13]; (28/52), 53.8% (39.5–67.8) [35]	(333/403), 82.6% (78.4–86.1)	Park [13], Gratzer [35]
	Secondary	(98/98), 100% (96.2–100)		
	Early latent	(41/41), 100% (90.7–100)		
	Late latent	(67/68), 98.5% (92.1–99.9)		
	Overall	(258/262), 98.5% (96.1–99.6)		

If the study distinguished specimens by treatment status, data for untreated patients are presented. For tests with only 1 published reference, sample sizes, and CIs are listed; otherwise, ranges of sensitivity/specificity estimates are listed. For sensitivity n/N represents positive test/true positives. For specificity n/N represents negative test/true negatives.

Abbreviation: CI, confidence interval.

### Aggregated Sensitivity

#### Primary and Secondary Syphilis

Among the manual assays, MHA-TP was less sensitive for primary syphilis (45.9–88.6%) than FTA-ABS (78.2–100%) or TP-PA (86.2–100%) [2–4,6–11,13,14,16,18–20,23,24]. For secondary syphilis, the sensitivity of MHA-TP was 90–100%, FTA-ABS was 92.8–100%, and TP-PA was 100% [2–4,7–11,13,14,16,19,23,24] (Table 2). Based on data from 2 studies that compared head-to-head test performance, FTA-ABS was less sensitive than TP-PA in both primary and secondary syphilis [11, 13].

A study by Park et al [13] found similar sensitivity for the ADVIA Centaur, Bioplex 2200, LIAISON, and Trep-Sure in primary syphilis compared with TP-PA and FTA-ABS; however, Gratzer et al [35] found poorer sensitivity of Trep-Sure in primary syphilis (54.8%, 39.5–67.8%). The Captia Syphilis G was 82.3–100% sensitive for primary syphilis in 3 studies, but sample sizes were small (6–13 cases) [17,30,32]. Overall, based on limited studies with small sample sizes, the sensitivity of the immunoassays in primary syphilis was comparable to the manual treponemal assays.

For the 5 treponemal assays that had data stratified by stage, all were 100% sensitive for secondary syphilis [13, 25, 34] (Table 3). Therefore, the sensitivity of the immunoassays was comparable to TP-PA and slightly higher than MHA-TP or FTA-ABS.

#### Latent Syphilis

Among the manual assays, sensitivity is similar among FTA-ABS, TP-PA, and MHA-TP for diagnosis of early latent syphilis (94.4–100%) and sensitivity was lower for late latent disease than early latent disease (84.5–100%) [2, 4, 11, 13, 17, 23] (Table 2). Two studies comparing the sensitivity of FTA-ABS versus TP-PA in late latent disease found conflicting results [11, 13].

Among the treponemal immunoassays with data by stage of syphilis (Table 3), sensitivity ranged from 95% to 100% for early latent syphilis and 91.7% to 100% for late latent syphilis [13, 17, 30, 32, 34].

#### All Stages Combined

Among studies of treponemal immunoassays that looked at overall sensitivity for all stages of syphilis combined, sensitivity ranged from 94.5% to 100% [12,15,21,22,25–29,31,33]. A single study that compared the overall sensitivity of 4 immunoassays (ADVIA Centaur, Bioplex 2200, LIAISON, TrepSure) found no statistically significant differences in combined sensitivity [13].

### Food and Drug Administration 510K Premarket Notification Data

Available premarket data submitted to the FDA for the treponemal immunoassays are presented in Table 4 [36–46]. Among the 13 immunoassays, 8 submitted sensitivity data stratified by stage (early and late latent syphilis combined). The clinical diagnostic criteria for syphilis diagnosis were not described in any of the 510k data. Three assays (Enzy-Well Syphilis IgG, Immulite 2000 Syphilis Screen, LIAISON) submitted data on positive percent agreement against a predicate assay in patients with “medically diagnosed” syphilis (clinical criteria not described); these demonstrated 97.9–100% agreement with the respective predicate assay [43, 45, 46].

**Table 4.** Sensitivity or Positive Percentage Agreement of Treponemal Tests for Diagnosis of Syphilis by Stage and Treatment Status [FDA Premarket 510(k) Data]

Assay	Test Performance		Reference
ADVIA Centaur	Patients with syphilis not analyzed separately		[40]
Architect Syphilis TP	Untreated (no 95% CI)	Treated (no 95% CI)	[39]
	Primary (25/25), 100% Secondary (27/27), 100% Latent (29/29) 100%	Primary (33/44), 75% Secondary (29/29), 100% Latent (25/25), 100%	
AtheNA Multi-Lyte T. pallidum IgG	Untreated	Treated	[42]
	Primary, N/A Secondary (40/43), 93.0% (80.8–98.5) Latent (6/11), 54.5% (23.4–83.3)	Primary (10/11), 90.9% (58.7–99.8) Secondary (39/39), 100% (92.6–100) Latent (45/52), 86.5% (74.2–94.4)	
	Congenital (2/3), 66.7% (9.4–99.2)		
Bioplex 2200 Syphilis IgG	Untreated	Treated	[44]
	Primary (10/12), 83.3% (55.2–100) Secondary (10/10), 100% (72.2–100) Latent (8/13), 61.5% (35.5–82.3)	Primary (15/16), 93.8% (71.6–98.9) Secondary (36/36), 100% (90.3–100) Latent (49/53), 92.5% (82.1–97.0)	
Bioplex 2200 Syphilis Total	Untreated	Treated	[36]
	Primary (25/26) 96.2% (81.1–99.3) Secondary (25/25) 100% (87.1–100) Latent (23/23) 100% (85.7–100)	Primary (25/29) 86.2%, (69.4–94.5) Secondary (26/26), 100% (87.1–100) Latent (27/27), 100% (85.1–100)	
Captia Syphilis-G Assay	N/A, see Table 3		
Elecsys Syphilis	Untreated	Treated	[37]
	Primary (25/25), 100% Secondary (25/25), 100% Latent (25/25), 100%	Primary (16/29) 55% Secondary (24/25), 96% Latent (25/25) 100%	
Enzy-Well Syphilis IgG	N = 125 pediatric and adult patients with syphilis, who were reactitve w/Captia Syphilis G; positive % agreement, all stages combined: (125/125), 100% (no 95% CI provided)		[45]
Immulite 2000 Syphilis Screen	N = 281 patients with “medically diagnosed” syphilis, n = 272 reactive with w/LIAISON; positive % agreement, all stages combined: 270/272, 99.3%, (97.4–99.9)		[43]
LIAISON	N = 51 patients with “medically diagnosed syphilis,” n = 48 reactive with Captia Syphilis G; positive % agreement, all stages combined: 47/48, 97.9% (89.0–99.9)		[46]
Lumipulse G TP-N	Untreated (no 95% CI)	Treated (no 95% CI)	[38]
	Primary (27/27), 100% Secondary (30/30), 100% Latent (183/200), 91.5%	Primary (2/2), 100% Secondary (25/25), 100% Latent (5/5), 100%	
Trep-Sure	N/A patients with syphilis not analyzed separately (see Table 3)		
Zeus Scientific T Pallidum IgG Test System	Untreated	Treated	[41]
	Primary, N/A Secondary (41/43), 95.3% (84.2–99.4) Latent (6/11), 54.5% (23.4–83.3)	Primary (11/11), 100% (76.2–100) Secondary (39/39), 100% (92.6–100) Latent (48/50), 96% (86.3–99.5)	

Abbreviations: CI, confidence interval; FDA, Food and Drug Administration; N/A, not applicable.

Among patients with untreated primary syphilis, sensitivity ranged from 83.3% to 100%; sample sizes ranged from 12 to 27 patients. For secondary syphilis, sensitivity ranged from 93% to 100%, with a sample size range of 10–43 patients. The AtheNA Multi-Lyte T. pallidum IgG (Zeus Scientific), the Zeus Scientific T. pallidum IgG Test System, and the Bioplex 2200 Syphilis IgG demonstrated poor sensitivity in untreated latent syphilis (54.5–61.5%); however, sample sizes ranged between 11 and 13 patients [41, 42, 44]. The other treponemal assays were 91.5–100% sensitive for latent syphilis (n = 25–200 patients).

### Aggregated Specificity

With the exception of 1 study demonstrating 87% specificity for FTA-ABS [15], the specificity ranges of FTA-ABS (92.0–100%) and TP-PA (94.0–100%) were similar, while MHA-TP ranged from 98.5% to 99.7% [2–5, 7, 8, 10–17] (Table 2).

The immunoassays demonstrated specificity ranging from 94.5% to 100% (Table 3), with the exception of TrepSure, which was 82.6% specific in a single study [13].

### Neurosyphilis

#### FTA-ABS

Thirteen studies described CSF FTA-ABS test performance (not all studies included both sensitivity/specificity) and were summarized in a prior systematic review [47]. In 3 studies of patients with definitive neurosyphilis (reactive CSF Venereal Disease Research Laboratory Test), the sensitivity of CSF FTA-ABS was 90.9–100% [48–50]. Among those with presumptive neurosyphilis where diagnosis was made based on reactive serology, other abnormal CSF indices, and clinical signs/symptoms, the sensitivity ranged widely (22.2–100%) [47]. A study by Luger et al [51] of 60 symptomatic patients defined neurosyphilis by comparing ratios of serum protein and CSF protein with a ratio of serum treponemal antibody and CSF treponemal antibody; in this study, the sensitivity of the CSF FTA-ABS was 100%. Another large study by Hooshmand et al [52] (n = 156) also found 100% sensitivity of CSF FTA-ABS, but a reactive CSF FTA-ABS was part of the case definition, thus the sensitivity results cannot be interpreted. The specificity of FTA-ABS varied greatly depending on whether true negatives were patients without syphilis or patients with syphilis, but not neurosyphilis. Six studies included patients without syphilis as true negatives, and the specificity of FTA-ABS was 100%; however, a study by Jaffe et al [53] found that CSF FTA was reactive in 5 of 15 patients with syphilis who had no other evidence of neurosyphilis. Eleven studies included patients with syphilis, but not neurosyphilis, and the specificity ranged from 55% to 100% [47].

For CSF TP-PA, 4 studies described test performance. A study by Castro et al [54] reported a sensitivity of 100% for the CSF TP-PA but the clinical characterization of true positives could not be interpreted given the data provided. The other 3 studies reported a sensitivity of 75.6–95.0%, with the highest sensitivities when using reactive CSF VDRL as the criterion for true positivity [54–57]. Specificities ranged from 85.5% to 100% and were highest if a titer of 1:640 or greater was used to define neurosyphilis [57]. Based on these limited data, CSF TP-PA appears to have similar performance to CSF FTA-ABS in studies with a mixed population of patients with definitive/presumptive neurosyphilis.

## Discussion

Among the numerous treponemal assays currently approved by the FDA, comparison of performance characteristics was more robust for the manual assays because there were few studies of the immunoassays that included clinically characterized specimens, stratified by stage. Among the manual treponemal assays included in this review (ie, MHA-TP, FTA-ABS, TP-PA), MHA-TP demonstrated poorer sensitivity for all stages of syphilis. Between the FTA-ABS and TP-PA, the 2 studies that compared their performance found lower sensitivity for the FTA-ABS for primary and secondary syphilis [11, 13]. Given the subjective nature of FTA-ABS interpretation, lack of quality control for FTA-ABS reagents, and the need for microbiologist experience, it is not recommended for use. TP-PA is the recommended assay among the manual treponemal tests.

Among the treponemal immunoassays, there were few published data on test performance stratified by stage, and sample sizes for Premarket FDA 510K data were small. There are insufficient data to distinguish differences in performance between treponemal immunoassays (eg, EIAs, CIAs, MBIAs) for laboratory diagnosis of syphilis. Of note, 2 studies found that TrepSure had poor sensitivity for primary syphilis [24] and significantly lower specificity than other immunoassays [13].

Several factors should be considered when interpreting these test performance data. Most studies were retrospective and used reactive serology as part of the inclusion criteria, which would bias sensitivity estimates towards 100%, particularly for primary syphilis. Studies utilizing previously banked specimens (both CDC and commercial serum banks) were included in this analysis, but the quality of staging/characterization of these specimens could not be assessed. For primary syphilis, it was unclear whether studies using banked specimens included dark-field positive-seronegative cases or just cases with reactive nontreponemal and/or treponemal serology. For latent syphilis, most studies combined early and late latent into a single category defined as “combined latent syphilis” or used a 2-year cutoff for defining early versus late latent disease. Some studies included prior treated cases and untreated (current) syphilis cases. When possible, our evaluation focused on untreated or current syphilis because the time between treatment and specimen collection was not described.

With regard to neurosyphilis, diagnostic criteria of the included studies were diverse and included various combinations of signs/symptoms with abnormal white blood cell count/protein and/or reactive CSF VDRL. As *T. pallidum* IgG can cross the intact blood–CSF barrier, reactive treponemal tests in the CSF are not specific for the diagnosis of neurosyphilis. Although the CSF TP-PA and CSF FTA-ABS demonstrated similar sensitivity and specificity, Harding et al found that a negative CSF treponemal test may not rule out neurosyphilis among patients with a high pretest probability (patients with syphilis and neurologic symptoms) [47]. Therefore, CSF treponemal tests have limitations with both sensitivity and specificity, and results need to be evaluated within the context of the clinical scenario, additional CSF testing (eg, VDRL, cell count, protein), and syphilis prevalence.

### Future Needs and Recommendations

1. Performance data are needed for the immunoassays using clinically characterized specimens, stratified by stage of syphilis. Studies should include sufficient numbers to stratify by HIV status so that performance among persons living with HIV can be assessed in the era of combined antiretroviral therapy. Many assays currently in use had no published data of this kind. This is particularly an issue with early primary and late latent disease.
2. Additional data are needed on the performance of treponemal tests in latent syphilis based on the CDC case definitions for latent syphilis [1].
3. Additional data are needed on the comparative performance of assays for diagnosis of neurosyphilis: CSF FTA-ABS, CSF TP-PA, and treponemal CIA/EIA in CSF.
4. Performance data are needed for the immunoassays (in serum) among patients with neurosyphilis.
5. There is a need to define serologic windows using modern treponemal and nontreponemal tests.

Some of these research and programmatic goals could be facilitated by the creation or resurrection of the CDC Syphilis Serum Bank with validated specimens, characterized by stage using standardized criteria, including seronegative, dark-field–positive primary syphilis specimens. This should also include specimens among patients without syphilis for specificity evaluations.

Other facilitators would include harmonization of criteria for evaluating performance of treponemal and nontreponemal tests, in particular characterization of true/false positives. Several newer immunoassays (eg, Elecsys Syphilis, Architect Syphilis TP, Lumipulse G TP-N) achieved this through a consensus of testing with a predicate immunoassay, plus RPR, plus TP-PA, where any 2 of 3 reactive specimens would be considered a true positive. More data are needed to determine whether this approach should become the common reference standard or predicate against which new immunoassays should be measured.

## Supplementary Data

Supplementary materials are available at *Clinical Infectious Diseases* online. Consisting of data provided by the authors to benefit the reader, the posted materials are not copyedited and are the sole responsibility of the authors, so questions or comments should be addressed to the corresponding author.

ciaa349_suppl_Supplement_Table

## Notes

***Financial support*.** This work was supported by the Centers for Disease Control and Prevention (I. U. P., Y. F., and L. P.). I. U. P. was supported by CDC (grant number 16IPA1611534-3).

***Supplement spon**sorship.*** This article appears as part of the supplement National Coalition of STD Directors, sponsored by the CDC.

***Potential conflicts of interest.*** The authors: No reported conflicts of interest. All authors have submitted the ICMJE Form for Disclosure of Potential Conflicts of Interest.
