# Supplementary material for: Sensitivity and Specificity of Treponemal-specific Tests for the Diagnosis of Syphilis
Source: Clin Infect Dis. 2020 Jun 24;71(Suppl 1):S13–20. doi: 10.1093/cid/ciaa349 (PMC7312216; doi:10.1093/cid/ciaa349)
Supplement: ciaa349_suppl_Supplement_Table [file ciaa349_suppl_supplement_table.docx]

|  |  |  |  |  |
| --- | --- | --- | --- | --- |
| **Reference #, Citation** | **Description of study type, design, population, and setting (gold standard)** | **Reported findings, quantitative results related to key question** | **Overall quality; strengths/weaknesses or limitations** | **Relevance to the key question and/or overall importance** |
| 1. (background, see references) |  |  |  |  |
| 2. Huber, T. W., et al. (1983). Journal of Clinical Microbiology **17**(3): 405-409. | Prospective, Univ hospital and PH laboratory, TX, US  Pts with DF+, first episode primary syphilis  FTA-aBS, RPR, MHA-TP, VDRL  **Gold standard**  **True pos: primary syphilis confirmed by + DF, chart review to determine first vs reinfection**  **True neg: n/a** | N=109 true pos  Sensitivity  FTA-Abs: 98.2%  RPR 92.7%  MHA-TP 72.5%  VDRL 72.5  In subset of 61 pts, reagent lots affetcted MHA TP sensitivity (50-70%, depending on reagent lot used) | **Clinically characterized and verified by darkfield and chart review**  Because MHA TP is not used very much the comparative data are not that useful but the standalone data in primary syphilis is useful  (unclear if reagent issue is relevant to this question—ask Sheila? | ***  FTA-Abs more sensitive than MHA-TP in primary syphilis |
| 3. Creegan (2007)  [Sex Transm Dis.](https://www-ncbi-nlm-nih-gov.ucsf.idm.oclc.org/pubmed/?term=creegan+2007) 2007 Dec;34(12):1016-1018. | Cross sectional, STD clinic, US  EMR queried to identify primary syphilis cases  Anyone with prior history of syphilis excluded  **Gold standard:**  **True pos: positive darkfield w treponemes**  **True neg: n/a** | N=51 patients with primary syphilis  Sensitivity  TPPA 86% (78-92)  VDRL 71% (61-79%)  RPR (same as VDRL) | **Clinically characterized primary syphilis using DF**  **Only new dx of syphilis considered, DF required for diagnosis**  Sensitivity only | ***  Sensitivity of TPPA to diagnosis primary syphilis better than VDRL/RPR |
| 4. Dyckman, J. D., et al. (1980). Journal of Clinical Microbiology **12**(4): 629-630. | Prospective, PH lab, TX, US  Specimens from pts with primary syphilis tested with VDRL, FTA-Abs and MHA-TP  **Gold standard:**  **True pos: DF + for T pallidum** | N=130 true pos primary syphilis  **Sensitivity**  FTA-ABS 91.5%  MHA-TP 82.3%  VDRL 68.5%  41 pts DF+ VDRL-  FTA-Abs 73% reactive  MHA-TP 51% reactive | **Clinically characterized sera, large number of pts with primary syphilis, DF confirmed**  Prior history of syphilis not described (but assume neg?) | ***  FTA-Abs is more sensitive that MHA-TP in early primary syphilis (VDRL about 70% sensitivity) |
| 5. Jaffe, H. W., et al. (1978). American Journal of Clinical Pathology **70**(2): 230-233 | Prospective, STD clinic and Univ Medical center, and VA, CO, US  Adult pts presenting for syphilis screening (not selected)  Comparing MHA-TP, TPHA, to FTA-Abs  True pos:  Primary DF positive. Chancre (DF- or not done) LAD and reactive serologic test, (at least 2 of 3) . Secondary syphilis: DF+ rash, mucous patch, or condyloma latum or clinical signs and reactive treponemal test  Latent syphilis—no sign of syphilis and reactivity in two treponemal tests  True neg— no history or clinical findings of syphilis A. Seronegative, BFP VDRL, single trep test reactive, or borderline FTA | N=68 pts with syphilis  N=935 without syphilis  Sensitivity (n),  MHA-TP/FTA-ABS  Primary (17) 76.4%/100%  Secondary (23) 100% (both)  Latent (27) 100% (both)  Specificity  MHA-TP 99.04%  FTA-Abs 98.7% | **Clinically characterized, stratified by stage, small numbers**  **Data presented as treated/untreated but due to small cell numbers data combined here.** | *** |
| 6. Byrne, R. E., et al. (1992). Journal of Clinical Microbiology **30**(1): 115-122. | Retrospective, diagnostic company lab, IL, US  Serum specimens from CDC serum bank. 3 different panels, and another panel from “other sources”  FTA ABS (DS) and western blot performed.  **Gold standard for true pos and true negative based on prior characterization by bank.** | Results from panel 1 and 3 (stratified by stage)  N= 75 from CDC panel (n=28 with syphilis)  FTA-ABS (n) sensitivity  Primary (9) 88.9%  Secondary (13) 100%  Latent (6) 83%  N= 90 from other sources  n=40 pts of these with primary syphilis  Sensitivity FTA-ABS 90%  Specificity (? Based on panels plus negatives from both panels and “other sources?”  92% | **Clinically characterized, and stratified by stage, but small numbers.**  **Also Unclear exactly how many true negatives there were as total number of specimens listed and numbers of true negatives in the table do not align.** | ***  Useful for FTA-ABS data by stage, but specificity data should be interpreted with caution as unable to reconstruct results from tables provided |
| 7. Van Eijk, R. V. W., et al. (1986). Sexually Transmitted Infections **62**(6): 367-372. | Retrospective, univ hospital, Netherlands  Frozen specimens from bank of pts with syphilis, and healthy blood donors  Fresh specimens from STD clinic patients (not used for test performance calculation)  Tested w TPHA, **FTA-Abs**, VDRL, TP-ELISA, AF-ELISA (not in use)  Gold standard:  True pos: clinical case definions  **Primary: lesions + TP in lesion or lymph node, and/or reactive serology**  **Secondary: TP in the lesions and reactive serology**  **Latent: reactive serology, no signs or history of past syphilis**  **NS: neuro symptoms, CSF FTA or TPHA and either CSF VDRL+ or CSF monoWBC >5 (or oligoclonal IgG)**  Old syphilis: treated according to GL and no evidence of reinfection  True neg: all blood donors assumed neg | N=253 pts with syphilis  N=500 blood donors    FTA-AbS  Sensitivity by stage, (n)  Primary (77) 93.5%  Secondary (44) 100%  Latent (35) 100%  NS (31) 100%  Old syphilis (66) 89.4%  Specificity 99.6% | **Clinically characterized sera, stratified by stage**  EL and LL combined into latent, all blood donors assumed to be negative (likely accurate given specificity >99%)    Large # w NS, (test performance of serum FTA-ABs not CSF tests) | *** |
| 8. Coffey, E. M., et al. (1972). "Evaluation of the qualitative and automated quantitative microhemagglutination assay for antibodies to Treponema pallidum." Applied Microbiology 24(1): 26-30. | Comparison of MHA-TP vs. FTA-ABS, VDRL, TPI.  Sera from 314 well-documented cases of syphilis based on clinical hx + lab data  172 biological false positives (VDRL pos and FTA-ABS and TPI neg)  22 narcotic users with pos VDRL and negative FTA-ABS  GOLD STANDARD:  314 = clinical case hx plus labs  51 = dark field | N = 314  MHA-TP = 64% % reactive for primary syphilis  96% secondary and early latent; 97% late latent; 98% late symptomatic  N = 51 (dark field pos)  53% positive for MHA-TP vs. 71% positive for VDRL and 78% positive for FTA-ABS  N=96  MHA-TP vs FTA-ABS  89/96 (93%) agreement  N=172 BFP  1.2% (2) reactive MHA-TP  N=22  21 MHA-TP neg (95% concordance) | Good comparison of MHA-TP vs. other treponemal tests. Many different types of samples were used in the comparison.  MHA-TP not used much in today’s testing. TPI is no longer utilized so data compared against this test is not useful. | ***  Provides some good data between various types of samples (BFP, narcotic users) and results (VDRL, FTA-ABS, TPI). Unfortunately, the MHA-TP is not used anymore and the TPI test is off the market. The comparisons therefore, are not relevant.  It does point out the weaknesses of the MHA-TP in identifying primary syphilis. |
| 9. Ijsselmuiden, O. E., et al. (1989). Journal of Clinical Microbiology **27**(1): 152-157. | Cross sectional, university hospital, Netherlands  Pts with syphilis from Univ hospital, pts with old syphilis, yaws samples in untreated children, blood bank specimens  Blood bank specimens tested with TPHA only  Others all tested with TPHA, FTA-ABS and VDRL  **Gold standard:**  **True pos: based on prior clinical diagnosis**  **True neg: TPHA neg** | N=148 pts with syphilis  N=53 old syphilis, VDRL+  N=114 old syphilis, VDRL-  FTA-Abs (n) Sensitivity  Primary (55), 84%  Secondary (39) 100%  Latent (54) 100%  Yaws (15) 93%  Treated syphilis  VDRL reactive (53) 98%  VDRL non-reactive (114) 83%  N=938 blood donors  Specificity 99.6% | **Clinically characterized sera, stratified by stage**  **EL and LL combined into latent**  **Includes yaws**  **Gold standard for specificity based on test that is no longer in use** | ***  Useful for sensitivity  FTA-Abs marginally less sensitive in primary syphilis |
| 10. Ijsselmuiden, O. E., et al. (1987)." European Journal of Clinical Microbiology **6**(3): 281-285. | Retrospective, STD clinic, Netherlands  Serum panel: pts known to have syphilis, (old and new) pts w/ other non-syphilis infections, healthy blood donors  Fresh screening samples from STD clinic (not included in calculations)  TP-ELIFA (not FDA), TPHA, FTA-Abs  **Gold standard:**  **True pos:**  **Primary: lesions + TP in lesion or lymph node, and/or reactive serology**  **Secondary: TP in the lesions and reactive serology**  **Latent: reactive serology, no signs or history of past syphilis**  **NS: CSF FTA or TPHA and either CSF VDRL+ or CSF monoWBC >5 (or oligoclonal IgG)**  **True neg: h/o and lab results give “no conclusive evidence of syphilitic infection”** | N=202 known syphilis (51 treated)  N=504 blood donors  N=104 (infections other than syphilis)  Sensitivity FTA-Abs  Primary (50) 90%  Secondary (43) 100%  Latent (47) 100%  NS (11) 100%  Treated syphilis (51) 100%  Specificity 99.6%  Also 2/104 pts with infections other than syphilis (RA, and measles) were FTA + | **Clinically characterized sera. Latent syphilis combined EL/LL. Sample size greater than 30 for all except NS**  Stand alone data for FTA useful but other tests not relevant | ***  FTA-Abs highly sensitive/specific except marginally less so in primary compared to other stages |
| 11. Larsen, S. A., et al. (1981) Journal of Clinical Microbiology **14**(4): 441-445. | Retrospective, CDC lab, US  Fresh sera from DeKalb STD clinic  Frozen sera from CDC VD Serology Lab Serum bank, previously characterized (1/2 of primary were DF+)  Tested with HATTS, MHA-TP, FTA-ABS, VDRL  **Gold standard:**  **True pos: previously characterized as syphilitic per bank,**  **True neg: 4 tests negative, 3 tests negative** | N=328 syphilitic sera  Sensitivity (n), MHA-TP, FTA-ABS)  Primary (79) 88.6%, 97.5%  Secondary (89) 100% (both)  Latent (103) 99% (both)  NS (10) 100% (both)  CV (21) 89.5%, 100%  Old syphilis (25) 100% (both)  N=592 true negatives (results for fresh first, then frozen sera)  Specificity (Fresh 191, Frozen 381) (20 excluded that were + on more than 1 test)  MHA-TP 99.5-99.7%  FTA-ABS 97.9-95.3% | **Clinically characterized sera, stratified by stage, large numbers of P and S syphilis**  **EL and LL combined into latent**  **True negatives did not include any clinical characterization**  Includes CV syphilis | *** |
| 12. Pope, V., et al. (1982). Journal of Clinical Microbiology **15**(4): 630-634 | Retrospective, CDC lab, US  Frozen syphilitic sera from CDC VD Serology Lab Serum bank, previously characterized (n=72)  Frozen “normal” sera, BFP VDRLs, and dz other than syphilis (n=32)  Fresh sera submitted to CDC VD lab from PH outpt clinic (routine PEs), blood donors from 2 banks (n=159)  Fresh sera from pts with syphilis from Houston HD, and ATL HD. (n not listed)  Tested w VDRL, FTA-Abs, MHA-TP, ELISA (not FDA approved)    **Gold standard**  **True pos: based on prior classification at bank**  **True neg: based on prior classification at serum bank.** | N=75 pts with syphilis  N=222 non syphilitic individuals  Overall sens/spec  FTA-Abs 100%/98.5%  MHA-TP 76%/98.5%    (FTA-Abs 100% for all stages)  Sensitivity by stage, MHA-TP (n)  Primary (24) 45.9%  Secondary (20) 90%  Latent (31) 90.3%  NS (3) 66.7%  CV (1) 100% | **Clinically characterized sera, stratified by stage**  **EL and LL combined into latent**    MHA TP performed more poorly than FTA-Abs, particularly in primary syphilis, but MHA-TP is not so widely used | *** |
| 13. Augenbraun, 1998  Sexually Transmitted Diseases **25**(10): 549-552. | Prospective, multicenter cohort study of early syphilis tx, US  Pts with clinical and lab evidence of syphilis (serology or DF/DFA+) enrolled and tx  2wk, 1, 2, 3, 6, 9 ,12 mo f/u  Pts had MHA-TP, FTA-ABS, RPR  **Gold standard:**  **True pos: Gold standard:**  **“clinical and serologic or microbiologic (Darkfield or DFA positive) evidence of Primary, secondary, EL or LL) either MHA-TP or FTA-ABS positive**  **True neg: n/A**  **plus either MHA-TP or FTA-ABS positive**  **True neg: n/A**  For pts with 12 mo f/up, Trep test seroreversions documnted | **N=525 total pts (**104 (20%) HIV+)  N=128 primary  N=243 secondary  N=139 EL  N=15 stage unknown  **MHA-TP**  Primary 88.6%  Secondary 98.8%  EL 100%  **FTA-ABS**  Primary 99.2%  Secondary 100%  EL 98.7%  N=261 w 12 mo f/up, % with either TST seroreversion =28, (11%)  12/261 (5%) MHA TP reversion  N=238 w two FTA results 12 mos apart22 (9%) FTA-ABS reversion | **Clinically characterized**  **Stratified by stage but then each test served as gold standard for the other**  **Unclear how initial serologic dx was made (RPR + either MHA-TP, or FTA???) not clear from methods**  **Large sample size for each stage**  **No specificity data** | *** |
| 14. Bosshard 2013  Journal of Infection **67**(1): 35-42. | Retrospecitve, university hospital, Switzerland, sera routinely sent to lab for syph diagnosis (syxs pts) and see true neg for controls  TPPA, VDRL,  Pathozyme, Euroimmun, recomWell (not FDA)  **Gold standard**  **True pos**: primary: anogenital/oropharyngeal chancre, or red lesion + serology, or asyxs contact to syphilis  Secondary—rash typical of syphilis  Latent—serology (VDRL/TPPA)  Tertiary—NS or cardiac syphilis  True neg: “negative sera without further specification” (n=50) or sera from pts with other infections  Borrella burgdorferi, CMV, Lupus, HIV, pregnant women, previous syphilis dx (n=30) | N=156 true positives  N=151 true negatives  Sensitivity TPPA  Primary (n=59)  100% (93.9-100)  Secondary (n=66)  100% (94.6-100)  Latent (n=25)  100% (86.3-100)  Tertiary (not calculated)  Specificity  99.2% (95.5-100) | **Clinically characterized sera**  **Stratified by stage, latents combined**  good numbers in primary and secondary stage  Unclear why TPPA specificity is so high if patients with previous syphilis are included as true neg.  Unclear how negatives were classified | ***  Perfect sensitivity and 99% specificity of TPPA in primary and secondary syphilis |
| 15. Manavi, K., et al. (2006). International Journal of STD & AIDS **17**(11): 768-771. | Prospective, STD clinic, UK  Pts diagnosed with syphilis based on clinical criteria, DF, initial serology  Murex ICE EIA, VDRL, TPPA  **Gold Standard: (true pos) Primary Lesion/chancre plus + serology**  **2ndary: Mucocutaneous syxs plus + serology**  **EL: + serology and neg serology in past 2 years, if no serology or >2 years, LL** | N=105 patients dx with syphilis  N=50 primary, 26 secondary, 8 EL, 21 LL  TPPA Sensitivity  Primary (50) 96%  Secondary: (26) 100%  EL (8): 100%  LL (21): 100% | **Clinically characterized sera stratified by stage**  No specificity data, definition of EL/LL differs from that in the US  Useful for TPPA data alone had high sensitivity for all stages of syphilis. the ICE EIA is not FDA approved | *** |
| 16. Lam, TK (2010)  International Journal of STD & AIDS **21**(2): 110-113. | Cross sectional, Social hygiene clinics (STD clinics), Hong Kong DOH  TP-PA, FTA ABS (other non-FDA approved assays)  Gold standard:  True pos: prior serology plus clinical symptoms  Primary: Chancre  Secondary: mucocutaneous signs  EL: no syxs, documented non-reactive serology in last 12 months  LL: no syxs, non reactive serology >12 months  True neg: “normal healthy subjects” | n=135 pts with syphilis   - n=39 primary - n=20 secondary - n=18 EL - n=58 latent unknown duration   n=43 pts without syphilis  **Sensitivity (TP-PA/FTAABS)**  Primary TP 94.9% (83.1-98.6%)  FTA 84.6% (70.3-92.8)  Secondary TP 100% (83.9-100)  FTA 95% (76.4-99.1)  Early latent 94.4% (74.2-99.0)  94.4% (74.2-99.0)  Latent unknown duration  91.4% (81.4-96.3)  84.5% (73.1-91.6)  **Specificity** 100% (91.8-100) for all | **Clinically characterized sera stratified by stage**,  Small numbers for both sensitivity and specificity calculation | *******  **Head to head test performance TP-PA, FTA-ABS, characterized by stage**  **FTA less sensitive in primary syphilis and latent of unknown duration.**  **Perfect specificity** |
| 17. Park, IU. Clin Infect Dis 2018 Jul 9. doi: 10.1093/cid/ciy558. [Epub ahead of print] | Cross sectional, Kaiser Permanente Northern/Southern CA, SFDPH lab  N=959 patients, 262 with current syphilis,  294 with prior syphilis only, 403 without syphilis. 7 treponemal tests performed, performance stratified by stage:   1. ADVIA Centaur CIA 2. Bioplex 2200 IgG MBIA 3. INNO-LIA 4. FTA-ABS DS 5. LIAISON CIA 6. TP-PA 7. Trep-Sure EIA   **Gold standard: clinical stage based on testing and chart review**  **Primary Syphilis**  chancre or lesions plus (1) + darkfield  microscopy plus reactive nontreponemal (stat RPR or lab VDRL) or treponemal serology,  or (2) negative darkfield (or darkfield not performed) with pos nontrep + Pos trep    **Secondary Syphilis**  mucocutaneous lesions  including presence of a rash (trunk, scrotum, palms/soles) and/  or patchy alopecia, mucous patches, and/or condyloma lata  with reactive nontreponemal and treponemal serology.  **Early Latent Syphilis**  absence of symptoms and  either (1) reactive nontreponemal and treponemal serology, or  (2) 2 reactive treponemal tests  no prior history of syphilis AND prior sexual contact to a case of early syphilis within past yr OR prior nonreactive serology within the past 12 months.  **Late Latent Syphilis**  absence of symptoms and (1)  reactive nontreponemal and treponemal serology, or (2) 2 reactive  treponemal tests, no prior history of syphilis, no serologic test results  in the prior 12 months, and no sexual contact to a case of early  syphilis in the prior 12 months.  **Prior treated syphilis** syphilis history documented in chart, but no signs or symptoms of syphilis and no  dx of syphilis in 6 months after specimen collection, also serofast pts  **True negative: No Syphilis, based on chart review and testing**  no dx of syphilis on the day of  testing or 6 months after the day of specimen collection,  no syphilis in the past medical history, no reactive prior syphilis  serology, and a negative  result in at least 4 of 7 treponemal tests | Sensitivity of FTA-ABS in primary syphilis was poor compared to the immunoassays and TPPA.  TrepSure EIA was less specific than the immunoassays or TPPA  Sensitivity (primary) (others see table 2)  ADVIA Centaur CIA 94.5% (84.9-98.9)  Bioplex 2200 IgG MBIA 96.4% (94.5-98.2%)  INNO-LIA 96.4% (94.5-98.2%)  FTA-ABS DS **78.2% (65.0-88.2)**  LIAISON CIA 96.4% (94.5-98.2%)  TP-PA 94.5% (84.9-98.9)  Trep-Sure EIA 94.5% (84.9-98.9)  Specificity  ADVIA Centaur CIA 95.5% (93.0-97.3)  Bioplex 2200 IgG MBIA 96.7% (94.4-98.2)  INNO-LIA 98.5% (96.8-99.5%)  FTA-ABS DS 98.0% (96.1-99.1)  LIAISON CIA 94.5% (91.8-96.5%)  TP-PA 100% (99-100)  Trep-Sure EIA 82.6% (78.4-86.1) | **Clinically characterized, stratified by stage**  Primary syphilis cases all had reactive serology (either trep or non trep positive), so sensitivity estimates for primary syphilis may be over estimates. | ******* |
| 18. Pope, V., et al. (2000). Journal of Clinical Microbiology **38**(7): 2543-2545 | Retrospective, national ref lab, US  Serum samples with known syphilis, diseases other than syphilis (DOTS), BFP non-trep tests  Test performance in treated/untreated  **Gold standard:**  **True pos: syphilis stage as characterized by serum bank**  **True neg:** DOTS and previous BFP (RPR, FTA-ABS)  Second study, 390 unknown samples, Captia, TPPA, MHAtp  Describes agreement between MHA-TP and combined Captia/TPPA results | N=100 pts with syphilis  N=100 diseases other than syphilis  N=50 BFP non treponemal tests  TP-PA Sensitivity (n) (range treated-untreated)  Primary (24) 87-89%  Secondary (50) 100%  Latent (26) 95-100%  Specificity 94% among pts w BFP  96 % in pts with DOTS)%  N=390 specimens  Overall agreement 96% for all 3 assays, any 2 assays 97% (96.9-97.2%) | **Clinically characterized sera for TPPA test performance,**  MHA TP, captia, and TPPA had high levels of agreement, but given no gold standard, test performance not calculated | ***  TPPA sensitivity slightly lower in treated pts, about 88% in primary disease |
| 19. Gratzer B (2014)  Sexually Transmitted Diseases **41**(5): 285-289. | Retrospective, STD clinic, IL, US  TrepSure, RPR, FTA-ABS  **Gold standard**  True pos: chancre/ulcer at least 1 pos syphilis serologic result plus No known history of syphilis  TS-EIA+ or Equiv/RPR+  TS-EIA+ or Equiv/FTA+  True neg: N/A | N=52 (51 MSM)  Sensitivity  TS-EIA 53.8% (39.5-67.8)  RPR 76.9% (63.2-87.5)  (RPR significantly higher p=0.005)  (sensitivity analysis excluding equivocals or making equivocals neg were similar) | **Clinically characterized** sera, large sample size for primary syphilis, sensitivity analysis to ensure equivocals didn’t bias outcome  No specificity data, no data for other stages | ***  Sensitivity of TS EIA poor in primary syphilis |
| 20. Young, H., et al. (1998). Journal of Clinical Microbiology **36**(4): 913-917. | Retrospective, PH STD laboratory, UK  Serum panel from pts with a known history of syphilis (both tx and untreated) n=101  Unselected screening specimens (n=1184)  All specimens tested with ICE EIA (non FDA) and Captia IgG  If reactive on either, then tested w FDRL, TPHA, FTA-Abs, and Captia IgM (not FDA)  **Gold standard:**  **For panel: prior serology, chart info on staging/treatment (EL are infection <2 years)**  **True pos: either EIA reactive x 2, and TPHA and FTA-ABS both positive**  **True neg: NEG ICE EIA and Captia** | N=105 panel and + screening specimens  N=1180 true negative screening speicmens  **Sensitivity 100% for both Captia/FTA-Abs in untreated syphilis for primary, 2^nd^, EL and 100% for treated LL**  **Sensitivity**  **Stage (n=treated/untreated)**  (n) CAPTIA/FTA-ABS  Primary (17/7)  C: 88.2-100% (same for FTA)  Secondary (21/2)  C: 90.5-100%  F: 85.7-100%  EL (9/2)  C: 88.9-100%/F: 77.8-100%  LL (19/12)  C: 100-91.7%/F: 100%  CV (1) 100%/100%  NS (8) 100%/100%  Unk (6) (not calculated)  transplacental ab (1) 100%/100%  Overall sensitivity n=105  Untreated (28) 92.4%/92.4%  Treated (75) 96.4%/100%  N=2 unknown  **Specificity**  **Captia/FTA-Abs**  **99.2%/99.9%** | **Clinically characterized stratified by stage, but small numbers in each stage**  **LL and EL definition cut off at 2 years instead of 1 year**  **Most untreated have cell number smaller than 5** | ***  Lower sensitivity in treated early disease may reflect seroreversion following antibiotic therapy |
| 21. Young, H., et al. (1998). International Journal of STD & AIDS **9**(4): 196-200. | Cross sectional, University hosp and GUM unit, UK  Unselected screening specimens, known specimen panel (syphilis at various stages, w known tx statu)  Tested with Syphilis Fast (non FDA), Captia IgG, VDRL  Any positive specimen was retested with all 3 tests  Additional cases found from screening specimens classified as tx or untreated  **Gold standard:**  **True pos: known positive specimen panel diagnosis OR Captia/VDRL pos**  **True neg: negative on all tests, (or pos screening neg confirmatory)** | N=114 true pos  N=1503 true neg  Captia Sens/Spec Overall  92.1%/99.2%  Repeat testing  94.7%/99.5%  Sensitivity (treated-untreated)  Primary( 8/6) 100%  Secondary (23/3)* 95.7%-100%%  EL (11/4) 90.9-100%  LL (19/13)* 94.7-92.3%%  CV (1) 100%  NS (10) 100%  UNK (15) (treatment status unk also)  Total n=114  Sens 94.7% | **Clinically characterized (according to prior panel results), stratified by stage**  **Sensitivity reported for each stage stratified by treated vs untreated, so sample size less than 5 in many cells** | ***  Captia test performance, high specificity, sensitive in primary syphilis, less so in latent syphilis |
| 22. Lefevre, J. C., et al. (1990). Journal of Clinical Microbiology **28**(8): 1704-1707. | Retrospective, University hospital, france  Specimens from pts untreated for syphilis (per chart review),  Neonates of mothers with tx syphilis, patients with treated syphilis  Tested with VDRL, 19S IgM-FTA-ABS, Captia Syphilis G, Captia Syphils M, TPHA/FTA  **Gold standard: True pos: Clinical characterization per chart review, plus MHA-TP AND FTA-ABS positive**  **True neg: N/A** | N=178 (9 samples tested twice)  N=96 untreated syphilis  N=63 with old syphilis  N=10 neonates  Captia syphilis G  Sensitivity  Primary (14/17) 82.3%  Secondary (13) 100%  EL (14) 100%  LL (33) 100%  NS (3) 100%  CS (1) 100%  Reinfection (15) 100%  Captia IgG positive for all pts with previously treated syphilis (72/72 specimens, n=63 patients), but 0/10 neonates whose moms were treated | **Clinically characterized, stratified by stage, small numbers**  **Some specimens tested twice, unclear rationale**  **No specificity data** | ***  Captia IgG high sensitivity by stage for all but primary syphilis and not + for neonates (? Did not measure passively transferred maternal antibody |
| 23. Marangoni (2005)  " Clinical & Diagnostic Laboratory Immunology **12**(10): 1231-1234. | 2 studies, both retro/ prospective univ hospital,and STD clinic, Italy  Retrospective study (specificity calculations) 2494 neagtaive controls from blood banks and 96 pts with possible BFP conditions, (sensitivity calculations) serum bank from pts with syphilis,  Prospective study—1800 routine unselected screening samples (used WB as gold standard, so not included here)  LIAISON, TPHA, RPR, WB  **Gold standard**  **true pos: per description had clinical and lab criteria c/w syphilis, using previously publishd criteria by Norris/Larsen**  **True neg: not stated, simple says the samples were “neg controls”** | N=131 clinically characterized sera  N=2590 “neg controls”  Overall Sens/spec  LIAISON 99.2/99.9  By stage:  Primary (7) 100%  Secondary (31) 100%  Latent (77) 96.1%  CV (5) 100%  NS (6) 100%  CS (5) 100%  LIASON gave no FP among pts with potential for BFP, and 0.12% of blood donors | **Clinically characterized sera stratified by stage but**  Small numbers in each stage, <10 except secondary  Latent not separated into early/late  Prospective study used WB as gold standard, so not included here)  Unclear how true neg were characterized (assumed all donors were neg) | ***  Only or one of few paper on Liaison sensitivity and specificity |
| 24. Wellinghausen, N. and H. Dietenberger (2011).  Clinical Chemistry & Laboratory Med **49**(8): 1375-1377. | Private lab Germany, study 1: prospective screening specimens, study 2: retrospective study from banked serum.  Screening tests: LIAISON CIA, ARCHITECT CIA, TP-PA  Confirmatory test: FTA-Abs and IgG/IgM immunoblot (recomBlot)  **Gold standard: Study 1**  True+: screening+ plus confirmatory trep test, and clinical characterized if both positive  True neg: consensus of screening panel  **Gold standard:** study 2  Clinically characterized serum bank from patients with prior syphilis | Study 1: n=577 (incl 318 prenatal)  18 true positives  (3 prim, 2 second, 7 EL, 6 LL)  Sensitivity  LIA: 100% (18/18)  ARCH: 100% (17/17)  TP-PA 100% (18/18), and Specificity  LIA 100% (558/558),  ARCH 99.8% (552/553), and TP-PA 99.6% (556/558)  Study 2: n=42 specimens, from 32 patients with syphilis  (6 prim, 13 second, 8 EL, 4 LL, 1 late NS)  Sensitivity 100% for all assays. (ARCH only 37/37 specimens due to volume) | **Clinically characterized**  Not stratified by stage due to small numbers  Retrospective study uses multiple specimens from the same patient in sensitivity calculation | **  Compares head to head performance of CIAs to traditional tests with small numbers of patients not stratified by stage. Almost perfect sensitivity/specificity for all assays |
| 25. Xu, M., et al. (2016). International Journal of Infectious Diseases 43: 51-57. | Cross sectional, university hospital, China  N=3326 subjects, (948 routine exams, 1358 outpts, 1020 inpts), all tested wth RPR and TPPA  Architect Syphilis TP, WB performed.  **Gold standard:**  **True pos: Clinical diagnosis (inc sexual history) + RPR/+ TPPA**  **True neg:** | N=256 pts with syphilis  n-632 “healthy subjects”  Architect TP Sensitivity/Specificity  97.27% (94.46-98.67)  99.61% (99.32-99.78) | **Clinically characterized, not stratified by stage, specificity data difficult to interpret since criteria for true negative are not described.** | ** |
| 26. Saral, Y., et al. (2012). Acta Dermatovenerologica Croatica **20**(2): 84-88. | University hospital, Turkey, retrospective  Architect CMIA vs RPR  **Gold standard:**  True pos: initial clinical diagnosis based on chart review and **TPHA +**  True neg: neg RPR, TPHA and CMIA | N=4109 true negatives  n=117 patients with suspected syphilis, 112 true positives  n=18 primary, 21 secondary (rest latent or old, results not stratified by stage)  Sensitivity 98%  Specificity 100%  PPV 100%  NPV 71% | **Clinically characterized, (+ lab standard for true positives.**  **Lab gold standard (consensus of panel) for true neg**  No confidence intervals provided  Not stratified by stage  True neg case definition includes neg Architect result so specificity would be perfect based on inclusion criteria | **  Architect sensitivity and specificity with partially characterized specimens, both >98% |
| 27. Marangoni, A., et al. (2009). "Laboratory Journal of Clinical Laboratory Analysis **23**(1): 1-6. | Retrospective, Univ hospital, Italy  Specimens from known syphilis patients (by stage)  Sepcimens from pts causing possible BFPs (lyme, pregnancy, CMV, mono, etc)  False pos Architect samples (Architect+, TPHA/RPR -)  Enzygnost, Architect, WB, TPHA/RPR  **Gold standard**  **True pos:** prior clinical/serologic evidence of syphilis  **True neg:** Architect neg, OR Architect + WB-/TPHA-/RPR- | N=244 patients with syphilis  N=74 (potential BFPs)  N=129 Architect+, TPHA-, RPR-  Architect  Sens: 99.2%  Specificity 98.4% (calculated with panel 2 (n=73) plus 8145 true neg sera screened during 3 month study period) | **Clinically characterized sera**  **Not stratified by stage** | **  Architect had >98% sensitivity and specificity even with specimens likely to cause BFP RPR |
| 28. XIA C. J Infect  Chemother (2018), https://doi.org/10.1016/j.jiac.2018.07.017 | **Prospective, University Hospital, China**  Elecsys syphilis CIA  Architect CMIA,  MIndray (Chinese assay)  Samples tested with all 3 assays, then TRUST, discordant results confirmed with TPPA  N=584 samples from unique patients  124 pregnant women, 452 with “various diseases”, 8 patients with syphilis  **Gold standard: RSS results plus signs/symptoms and past medical history**  **True positive: 1,2, or 3 CIA reactive/TRUST reactive,**  **1,2,or 3, CIA-reactive, TRUST-non-reactive, TPPA reactive**  **Known h/o syphilis**  “Patients who recently or previously  infected with Treponema pallidum were also diagnosed with syphilis”  **True negative: 3 CIAs non-reactive**  **1,2,3, CIA-reactive, TRUST-non-reactive, TPPA non-reactive** | Elecsys syphilis  Sensitivity: 100%, 57/57, (93.9%-100.0%)  Specificity: 98.5%. 519/527  Architect  Sensitivity: 100%, 57/57, (93.9%-100.0%)  Specificity 94.5%, 498/527 (92.2%-96.3%)  For 14/57 with known stage  3 primary  5 secondary  1 tertiary  5 latent  All three CIA and TRUST were reactive | **Clinically characterized, not stratified by stage because most had stage unknown.**  Clinical characterization not well defined. “recently or previously infected with T pallidum” so old and new syphilis were likely mixed together? | **  Little data on the Elecsys outside of 510K so useful from that standpoint |
| 29. Silletti, R. P. (1995). Journal of Clinical Microbiology **33**(7): 1829-1831. | Cross sectional, Public hospital lab, NY, US  Routine screening specimens (STD clinic specimens excluded), prenatal specimens  All specimens tested w/ RPR, Captia IgG and Captia IgM, then FTA-ABS performed on all. Chart reviews performed for all.  **True pos: evidence of syphilis diagnosis in chart AND specific therapy given, OR no active disease per chart review but RPR+ FTA-ABS reactive, OR CAPTIA IgG+, RPR-, Captia IgM+**  **OR Captia IgG/IgM neg, RPR+/FTA+**  **True neg: no evidence of syphilis in chart and RPR, FTA/Captia IgG-**  **BFP RPR (Captia IgG-, FTA-ABS-, Captia IgM neg)** | N=911 specimens met criteria  Captia Sens/spec/PPV/NPV  100%/98.2%/78.9%, 100%  (screening)  N=46/646 (7%) syphilis prevalence  Captia sens/spec  100%/97.8.%  (prenatal)  N=10/265 (4%) syphilis prevalence  Captia sens/spec  100%/99.2% | **Clinically characterized data, both negatives and positives had chart review**  **Not stratified by stage, small numbers, particularly in prenatal pts** | ** |
| 30. Cole, M. J., et al. (2007). European Journal of Clinical Microbiology & Infectious Diseases **26**(10): 705-713. | Cross sectional, blood bank and commercially specimen bank, and PH lab bank, UK  Unselected blood donors, banked specimens with known clinical history/staging  15 treponemal tests, but only TPPA and Captia FDA approved and in use (4 TPHA assays but not currently in use)  **Gold standard:**  **True pos: based on known prior disease stage/treatment status**  **True neg: not specified (assume all blood donors would be neg or at least no h/o syphilis)** | **Sensitivity**  Captia 94.7% (88.8-98)  TPPA 99.1% (95.2-100)  **Specificity**  Captia 100% (98.5-100)  TPPA 100% (98.5-100) | **Clinically characterized**  **Most tests not FDA approved**  **Not stratified by stage** | **  Captia and TPPA have perfect specificity, TPPA marginally better sensitivity |
| 31. Young , H., et al. (1995). International Journal of STD & AIDS **6**(2): 101-104. | Retrospective, GUM clinic, UK  Selected pts with known h/o prior + syphilis serology  Stages described but results not stratified by stage  Sensitivity of VDRL, TPHA, FTA-ABS, Captia IgG HIV+ vs HIV neg  **Gold standard:**  **True pos: prior reactive syphilis serology and clinical diagnosis**  **True neg: n/a** | N=59 pts w prior syphilis  N=28 HIV+, n=31 HIV-  Sensitivity (HIV+)  FTA-ABS 79%  Captia IgG 82%  Sensitivity (HIV-)  FTA-ABS 97%  Captia IgG 97%  FTA-Abs more sensitive in HIV- (p<0.05), captia NS | PRIOR Syphilis Only  **Clinically characterized, not stratified by stage**.  **No specificity data**  **Looking at prior syphilis only, not current syphilis, and prior syphilis history unknown**  **Numbers too small to stratify by stage of syphilis or CDC stage of HIV infection** | FTA-Abs and to a lesser extent EIA positivity may serorevert after treatment in patients with HIV  (Background only) |
| 32. Romanowski, B., et al. (1987). ."Sexually Transmitted Diseases **14**(3): 156-159 | Prospective, PH clinics, AB, Canada  N=128 pts with anogenital lesions  Tested with TP monoclonal antibody,  RPR, MHA-TP, and FTA-ABS  **Gold standard:**  **True pos: positive DF, and/or newly reactive serology, OR a four fold greater increase in quantitative RPR**  **True neg: DF negative, but unclear how serologic criteria may also have been used.** | N=66 pts with syphilis (63 primary, 3 secondary)  N=62 patients without syphilis  Sensitivity/specificity for P/S syphilis  FTA-Abs  92%/87% | **Clinically characterized, not stratified by stage but most of cases were primary syphilis.**  **Characterization of true negatives is unclear, and so difficult to interpret specificity** | ** |
| 33. Moyer, N. P., et al. (1984). Journal of Clinical Microbiology **19**(6): 849-852. | Prospective, univ hospital, IA, US  Tested with FTA-ABS and HATTS and VDRL  **Gold standard:**  **True pos: FTA-Abs pos and/or history of syphilis in chart, confirmed against health dept records**  **True neg: no history of syphilis in chart** | N=123 true pos  N=368 true neg    Sensitivity/Specificity  FTA 98.4%/96.2% | **Clinically characterized sera**  Data were stratified by stage for those with information but cell sizes small and half had stage unknown. (n=61) so only overall data presented here | ** |
| 34. Liu, C., et al. (2014). Journal of Clinical Laboratory Analysis **28**(3): 204-209. | Prospective, university hospital, China, screening population and pts with syphilis  TRUST, RPR, TPPA, TP-CMIA (Architect), TP-ELISA (Xiamen)  **Gold standard:**  Pos: signs/symptoms of syphilis, “latent infection” (not sure how characterized)  Neg: asymptomatic, “not at risk for syphilis”, two neg treponemal tests  (pre-op screening or PE) | N=210 true neg  N=160 true pos  Sensitivity/Specificity  TPPA 96.25%/100%  ARchitect 100%/90.95% | **Clinically characterized sera**, using combination of clinical and lab data (for neg)  Not stratified by stage  Unclear how latent syphilis was characterized | **  Architect more sensitive but less specific than TPPA in clinically characterized specimens (although accuracy of classification is uncertain) |
| 35. Farshy, C. E., et al. (1983). Journal of Clinical Microbiology **17**(2): 245-248. | Prospective, STD clinic, GA, US  STD clinic pts with clinically dx syphilis (criteria not described), and other pts presenting for screening  Two different reporting systems (DS reporting system downgrades initial borderlines that are repeat neg to NEG)  FTA-Abs DS vs FTA-Abs  **Gold standard**  **True pos: clinical dx of syphilis (criteria not described)**  **True neg: Not described** | N=35 true pos  N=311 true neg  Sensitivity/Specificity  FTA-Abs DS 94%/96%-98% (if using DS reporting method)  FTa-Abs 91%/93%  (Difference in sensitivity due to reporting methods) | **Clinically characterized but not stratified by stage and dx criteria unclear. Unclear how true neg classified**  Unclear if DS vs conventional fTA is relevant. differences in performance noted may be affected by the DS reporting system | ** |
| 36-47 (FDA data see references) |  |  |  |  |
| 47. Harding (2012)  Sexually Transmitted Diseases **39**(4): 291-297 | Systematic review  CSF tests were  FTA-ABS, FTA, TPHA, MHA-TP    Sensitivity, specificity (S+, NS-), specificity (syphilis neg), NPV  **Gold standard: Clinical criteria alone, clinical plus abnormal CSF WBC, clinical OR + CSF VDRL, positive CSF VDRL, +CSF VDRL, OR >5wbc OR protein >45** | 18 studies,  40 measures of sensitivity  30/40 had sensitivity ≥80%  studies using + VDRL as criteria demonstrate nearly 100% sensitivity.  21 measures of NPV. 18/21 demonstrated NPV >90%  3 measures NPV was 58-79%  In the general population, a neg CSF treponemal tests effectively rules out NS.  NPV varies depending pre-test probability. In usual clinical setting for these assays, pt has high probability of NS (sign/syxs) of syphilis, so the NPV would be lower than in gen population | Comprehensive data on sensitivity, specificity, NPV up until 2006 so needs update of last 10 years  Gold standard: NS diagnosis highly heterogeneic. | ***NS  Systematic review of test performance of treponemal tests using heterogeneous definitions of NS, and so no uniform gold standard |
| 48. (Birry et al see summary table in Harding et al. #47) |  |  |  |  |
| 49. Marra (2004)  Neurology **63**(1): 85-88. | Prospective, univ hospital, WA, US  HIV+, reactive non-trep+trep, no h/o of NS  HIV+, with no h/o syphilis, and neg RPR  CSF VDRL, WBC, FTA-ABS, FTA-DIL, (phosphate buffered saline sub for sorbent) FTA  Gold standard:  **NS pos: CSF VDRL+**  **Equivocal: WBC>5, CSF VDRL-**  **NS neg: CSF WBC <5 and CSF VDRL-,** | N=47 pts with syphilis--2 primary, 25 secondary, 8 EL, 8 LL, 4 unk  CSF results--  N=7 NS  N=19 equivocal  N=21 normal CSF  Sensitivity/Specificity  FTA-ABS 100%/71%  FTA-Dil 86%/67%  FTA 100%/43% | **Clinically characterized study entry but NOT for NS. For NS, lab criteria only.**  **All enrolled in study of CSF abnl in syphilis, so ? all neurologically asymptomatic (not congruent with current GL)** | ***NS  CSF FTA and FTA-ABS sensitive in pts with +VDRL |
| 50. Marra, C. M., et al. (1995).  Archives of Neurology **52**(1): 68-72. | Prospective, univ hospital, WA, US  Patients with untreated primary secondary syphilis underwent LP with paired serum specimen  CSF MHA-TP and FTA-ABS  NS definitions  Definitive: CSF VDRL positive  Possible (elevated WBC >5 or protein >45 | N=8 definitive NS  N=11 possible NS  CSF FTA-ABS  100% for definitive NS (7/7)  70% for possible NS (7/10) | **Clinical characterized cases of definitive and possible NS.**  **CSF VDRL required for definitive NS diagnosis, not all specimens had sufficient volume for all tests** | ***NS  Sensitivity of FTA-ABS is lower in possible/presumptive NS than in cases with + CSF VDRL |
| 51 and 52 (see summary table in Harding, et al #47) |  |  |  |  |
| 53. Jaffe, H. W., et al. (1978). Archives of Internal Medicine **138**(2): 252-255. | Both retrospective pts and prospective pts included, VA and public hospital, GA, and private hospital, MN, US  Pts having spinal anesthesia, pts having diagnostic LPs (any reason), for Mayo, MN, pts with reactive CSF FTA  CSF FTA (? In use) **CSF FTA-Ab**s and CSF MHA-TP (not in use), CSF VDRL  **Gold standard:**  **True pos: serum reactive for FTA-or MHA-TP (no clinical signs, neg CSF VDRL and normalCSF indices)**  **For CSF FTA-Abs analysis, prior known CSF-FTA pos**  **True neg: serum negative VDRL, FTA, and MHA=TP** | N=15 latent syphilitics  N=29 pts with prior + CSF FTA  N=177 non syphilitics  5/15 pts with latent syphilis had + CSF FTA  CSF FTA-Abs performance:  Among pts with prior CSF FTA+, correlation of FTA-Abs  (Sensitivity) was 45%  Specificity (177) 99.44% | **Laboratory criteria as gold standard, mostly descriptive, is CSF FTA even performed anymore or is it just FTA-Abs? May not be relevant** | *  If CSF FTA is not a legitimate gold standard then possibly exclude |
| 54. Castro, R., et al. (2006). Journal of Clinical Laboratory Analysis **20**(6): 233-238. | Retrospective, community and univ hospital, Portugal  Inpatients with reactive serologic tests for syphilis, pts with neurologic infections and neg serology  CSF VDRL, MHA-TP, TPPA, FTA-ABS  **Gold standard**  **True pos: Serum + FTA-abs, TPPA MHA-TP and > 10 WBC,**  **+/- CSF VDRL**  **+/- clinical symptoms**  **True neg: negative VDRL, serum MHA TP FTA, other neuroinfection** | N=18 pts with NS (16 pts with + CSF VDRL both syxs and asyx) 2 with syxs, >WBC and neg cSF VDRL)  N=46 patients with non syphilis neuro infections  Sensitivity/specificity  CSF FTA ABS 100%/100%  CSF TPPA 100%/100%  10/57 patients with prior treated syphilis had at least 1 abnormal CSF treponemal test  29/67 pts with latent syphilis had at least 1 trep test abnormality  19/67 pts with latent syphilis and normal WBC had 1 trep test abnl | **Clinically characterized**  **True neg**  **includes asyx and syx NS**  state that +VDRL CSF was part of gold standard but CSF FTA-and TPPA both pos for pts w neg VDRL but >WBC-CSF  Head to head comparison of CSF MHA TP, TPPA, FTA ABS but difficult to interpret test performance bc in tables some patients with syphilis (not classified NS) have abnl CSF WBC, + VDRL (meet case definition yet not included as such)  Tables challenging to interpret | ***NS  CSF treponemal test performance in pts with strictly defined  NS and non-syphilis neuro infections. 100% sensitivity/specificity |
| 55. Guarner, J., et al. (2015). American Journal of Clinical Pathology **143**(4): 479-484. | Retrospective, university hospital, pts eval for NS  CSF VDRL, TrepSure, TPPA, (INNO-LIA, MaxiSyph)  **Gold standard:**  **True pos:** VDRL positive plus symptoms c/w NS (11)  Symptoms but neg CSF VDRL (2)  Reactive CSF VDRL and (no history) (1)  **True neg:** pts who had a CSF VDRL performed during same week as reference group | N=14 in reference group  N=18 controls  **Sensitivity**  CSF VDRL-85.7%  TrepSure EIA-92.9%  TPPA 83.3%  **Specificity** 100% for all assays  **PPV** 100% for all  **NPV**  CSF-VDRL 89.5%  TrepSure 94.7%  TPPA 90% | Head to head test performance **mostly** **clinically characterized** ***sera*** (13/14)  Small numbers, lack of uniform gold standard | ***NS  TrepSure EIA and TPPA in CSF more sensitive than VDRL.  Useful adjunct to Harding/Ghanem 2012. |
| 56. Zhang, H. L., et al. (2013). Dermatology **226**(2): 148-156. | Retrospective, University hospital, all inpatients, HIV neg, median age 50  Inclusion: NS based on CDC GL. Confirmed NS: clinical dx of syphilis + CSF VDRL  Presumptive: clinical dx of syphilis and NR VDRL, with CSF >10 WBC, or protein >500 mg/l and neurologic signs/syxs  Excl: no HIV test, HIV+  **Gold standard:** clinical characterization plus laboratory findings | N=149 patients with NS  (124/149, 83% symptomatic, 17% asyx)  CSF TPPA  149/149 serum TPPA+  134/149 (89.9%) + CSF TP-PA reactive  88/149 (59.1%) CSF pleocyt  84/149 (56.4%) elev protein  37 (24.8%) had normal WBC and protein | **Clinically characterized (inc also lab gold standard using CDC criteria for dx**  Unclear why asymptomatic NS patients had CSF eval in first place—not clear if pts with other reasons for abnl CSF were excluded  (adjunct to Harding/Ghanem 2012) | *****NS**  Describes sensitivity almost 90% of CSF TP-PA in clinically characterized sera |
|  | | | | |
| **Test Performance Studies of Lower Relevance (e.g., laboratory gold standard only)** | | | | |
| **Reference #, Citation** | **Description of study type, design, population, and setting (gold standard)** | **Reported findings, quantitative results related to key question** | **Overall quality; strengths/weaknesses or limitations** | **Relevance to the key question and/or overall importance** |
| Atkas G et al (2007)  International Journal of STD & AIDS **18**(4): 255-260. | Retrospective, university medical center, Turkey, screening pop  FTA ABS, TPPA, ICE, Enzywell TP, IgG+IgM, Captia Syph M, WB (only FTA/TPPA FDA approved)  **Gold standard: FTA pos or neg**  **(additional tests used to characterize FTA neg as FN)** | N=94 FTA pos, n=25 FTA neg  N=3 equivocal  TPPA vs FTA ABS  97.8% pos agreement  92% neg agreement  Among other assays, % agreement FTA vs other trep tests was 95.9-98.3%  Found 2 neg FTA that were + on 5 other trep tests | **Laboratory gold standard, single assay**  Authors conclude that FTA more prone to give equivocal and FN results than other trep tests | * |
| Becker (1976) Late Syphilis; Arch Otolaryngol  102:729-731, Dec 1976 | Patients who reported to one ENT (author) over a 12-month period at UCLA with otitic symptoms with reactive FTA-ABS test during late syphilis. N=21 | SENS  FTA-ABS = 98%  TPI = 90%  Non-trep = 73%  9% discordance b/t FTA-ABS and TPI | Study examines FTA-ABS performance in late syphilis  FTA-ABS is compared to the TPI, which no longer is in use | *  Useful information for FTA-ABS performance in late syphilis, however, no comparison between another trep test can be made since TPI is no longer in use. |
| Binnicker (2011)  Journal of Clinical Microbiology **49**(4): 1313-1317. | Cross sectional, national reference lab, MN, US  Samples previously tested for syphilis, consecutive samples from national reference population  FTA (not ABS), Bioplex 2200 IgG  TPPA, TrepChek EIA, TrepSure  TrepID, ViraBlot IgG, IgM Wb  **Gold Standard: 1) FTA**  **2) consensus of test panel (≥4/7)** | N=303 samples  N=100 (prev tested for syph)  N=203 (general ref pop)  **FTA Gold standard**  **Sensitvity**  Bioplex 96.9% (90.9-99.3)  TPPA 95.9% (89.5-98.7)  Trep Chek 95.9% (89.5-98.7)  TrepSure 96.9% (90.9-99.3)  **Specificity**  Bioplex 98.5% (95.6-99.7)  TPPA 97.6% (94.3-99.1)  Trep Chek 98.5% (95.6-99.7)  TrepSure 94.7 (90.6-97.1)  **Consensus of test panel**  **Sensitivity**  Bioplex 100% (95.3-100)  FTA 100% (95.3-100)  TPPA 98.9% (93.6-99.9  Trep Chek 98.9% (93.6-99.9)  TrepSure 98.9% (93.6-99.9)  **Specificity**  Bioplex 98.6% (95.7-99.7)  FTA 98.6% (95.7-99.7)  TPPA 97.6% (94.3-99.1)  Trep Chek 98.6% (95.7-99.7)  TrepSure 94.3 (90.1-96.8)  **TAT in hours**  Bioplex 1.75  FTA 3.3  TPPA 4.0  Trepchek/Trepsure 2.2-2.3 | **2 different laboratory gold standards**    Unclear if 100 samples were supposed to be positive (94/100 were with FTA and consensus of panel-) | *  All four assays had similar performance regardless of gold standard, TrepSure was marginally less specific.  Bioplex had shortest TAT, TPPA longest |
| Buono, SA. Diagnostic Microbiology and Infectious Disease, 89 (2017) 173–177 | Prospective, urban public health laboratory  N=1000 screening specimens  ADVIA Centaur CIA, LIAISON CIA, Trep-Sure EIA, RPR  FTA-ABS was performed to help adjudicate discordants  **Gold standard: TP-PA**  **True pos: TP-PA positive**  **True neg: TP-PA negative**  **Used surveillance data to characterize discordant specimens only,**  **Compared traditional to reverse sequence algorithm** | Centaur:  Sensitivity: 98.7, 149/151, (95.3–99.8)  Specificity: 99.8, 834/836 (99.1–100)  LIAISON:  Sensitivity: 98.7, 150/152 (95.36–99.84)  Specificity: 100, 834/835, (99.56–100)  TS-EIA  Sensitivity: 97.4, 147/151, (93.4–99.3)  Specificity: 100, 830/831, (99.6–100)  RSS: 151 seropositive  Traditional: 113 positive  Of 38 discordant samples  (RPR-negative, TP-PA/CIA/EIA-positive).  26 previous history of syphilis,  11 did not have a previous  history of syphilis, and  1 known BFP for trep testing | **Single laboratory test as gold standard, only characterized discordant specimens**  Also useful information on TAT for (RSS vs Traditional algorithm) (Table 4, not included in this summary but included in paper | *****  **All 3 CIA/EIAs were similar to TP-PA** |
| Busse, C., et al. (2013). Clinical Laboratory **59**(5-6): 523-529. | Retrospective, University hospital, Germany  Prenatal screening, suspected syphilis pts, pts with borreliosis or HIV, syphilis serum panels  Virotech (not FDA)  TrepSure EIA (TS-EIA)  TPPA/FTA-ABS (combined)  **Gold standard:**  **True pos: TPPA and FTA ABS reactive**  **True neg: TPPA and FTAABS neg** | N=183 true neg  N=218 true pos    Sensitivity (lab GS)  TS EIA 100% (98.3-100%)  Specificity  TS EIA 93.9% (89.4-96.9%)  S/CO of 12 correponds or greater corresponds to TPPA titer of 1:80 or greater. | **LAB gold standard (consensus of 2 tests)**  **Mentions also n=20 specimens for analytical sensitivity and n=74 specimens from serum bank but no results for 74 specimens** | *  TS EIA perfect sensitivity, 94% specificity using lab gold standard, background data on S/co TS-EIA of 12 corresponding to high TPPA Titer. |
| Castro R et al (2001)  American Journal of Clinical Pathology **116**(4): 581-585. | Prospective, university hospital (in patients) and STD clinic, Portugal  Patients given therapy for early syphilis  RPR, MHATP, FTAABS, TPPA  Repeat serology, 1, 2,3,6, 12 months. S/S of TPPA  **Gold standard: (true pos)**  **MHA TP (analysis 1)**  **Or FTA-ABS (analysis 2)**  **Separate analysis among pts with primary syphilis (+MHA was gold standard for FTA, and vice versa)**  **True neg: MHATP- (analysis 1)**  **FTA-ABS – (analysis 2)** | N=449 total patients  (MHA as GS)  Sensitivity 100%  Specificity 94.4%  (FTAABS as GS)  Sensitivity 98.5%  Specificity 100%  n-28 primary syphilis  Sensitivity  TPPA 100%  FTAABS 89.2%  MHA TP 89.2%  N=54 returned for f/up, at 12 mos 0 seroconversions of TPPA or MHATP (FTA not described) | **Mostly single lab test as gold standard. Limited clinical classification to: “suspected of having early syphilis”**  **Clinical classification of primary syphilis unclear (described as serology “revealed” primary syphilis)**  High loss to follow up (89%) | * (b/c classification unclear)  Test performance of TPPA very high, using other manual trep tests as gold standard,  ?useful data for primary syphilis  descriptive data on seroconversion after treatment |
| Dans, P. E., et al. (1977). "The FTA-ABS test: a diagnostic help or hindrance?" Southern Medical Journal 70(3): 312-315. | 4,750 patients from CO General Hospital VD clinic from 2/73 to 4/74 (14 months) with 1)hx of prior syphilis 2)sore/rash 3) recent contact with syphilis infected person 4) reactive syphilis serology.  Sera from 1043 patients sent to lab for testing.  VDRL, FTA-ABS, MHA-TP performed at local lab ad CDC | N=395 serum samples from 226 patients tested at both labs  97.5% agreement b/t labs for VDRL testing  MHA-TP 71/76 positively identified syphilis correctly | Clinically characterized, not stratified by stage  Comparison of FTA-ABS to MHA-TP.  A lot of data presenting inter-laboratory testing results vs. direct comparison of tests | *  Discussion of weakly positive FTA-ABS results in a low prevalence population as potentially problematic.  Recommends that FTA-ABS only be used with VDRL positive results or for late syphilis only. |
| Gomez E (2010)  Clinical & Vaccine Immunology: CVI **17**(6): 966-968. | Prospective, university hospital, MN, US, general screening population  Bioplex 2200 IgG and separate IgM (MFIA),  TrepChek IgG/IgM  TP-PA, RPR (for discrepancies)  **Gold standard:** (initially TrepChek results, but then further testing of discrepants)  **True pos:** TC-EIA+/MFIA+  MFIA+/EIA-/TPPA+ or MFIA IgM+  **True neg:** EIA neg/equiv and Bioplex neg | N=1008 specimens  Bioplex IgG sensitivity  98.7% (77/78, 92.1-99.9)  Specificity 98.5% (653/663, 97.5-99.1)  N=671  Bioplex IgM Sensitivity  100% (8/8)  Specificity 98.5% (653/663) | **Consensus of 2 lab tests as gold standard**  Not all discrepants tested with IgM MFIA, sample size <10, for sensitivity analysis  TrepChek no longer on market and replaced with TrepSure (specificity issues with TrepChek per CDC MMWR) | *  Bioplex MFIA IgG or IgM was >98% sensitive/specific using lab tests as gold standard |
| Hooper, N. E., et al. (1994). Clinical & DiagnosticLaboratory Immunology**1**(4): 477-481. | Cross sectional, single state commercial lab MD, US  Routine screening specimens Tested with RPR and Captia IgG  Reactive samples tested with fTA-ABS, MHA-TP, selected samples for Captia IgM  Chart review done for EIA+/RPR- and to determine treatment status (does not state that all true pos had chart reviews  **Gold standard**  **True pos: + RPR and FTA-ABS (some chart review done for treatment status)**  **True neg: negative in both Captia and RPR OR BFP RPR (determined by f/up trep testing)**  Describe technical difficulties w EIA index values. initial and final results presented after resolution (final results presented | N=1000 specimens,  N=34 with syphilis diagnosis  N=961 with no syphilis diagnosis  N=5 inconclusive  N=31 not previously treated pts with syphilis  Sensitivity/Specificity  Captia 100%/999%  RPR 86.1%/99.4% | **not stratified by stage** Level of clinical characterization unclear. Discordants had chart review and treatment status known but does not state that all true pos pts had clinical characterization.  Rating based on assumption that some chart review done on all 31 to determine tx status  **Place under lab gold standard b/c clinical characterization not clear** | *  Captia more sensitive than RPR in dx of untreated syphilis |
| Hughes, G. B. and I. Rutherford (1986). Annals of Otology, Rhinology & Laryngology **95**(3 Pt 1): 250-259. | Case-control, univ hospital, OH, US  Test performance of FTA-ABS vs RPR  **Gold standard:**  **True pos: otosyphilis**   1. **Inner ear dysfxn (with or without other syphilis syxs** 2. **+ FTA-ABS (+/- RPR titer)**   **True neg: Controls**   1. **No h/o of syphilis, neg PE** 2. **Hearing loss (bilateral) c/w presbycusis no dizziness or hydrops** | N=31 otosyphilis cases (25 acquired, 6 CS) out of 5439  570/100K (prevalence among those with any hearing complaints)  Sens/Spec/PPV/NPV  FTA-Abs  100%/98%/22%/100%  RPR  55%/97%/9%/99% | **Clinically characterized, but FTA-ABS was required for diagnosis, so sensitivity would be 100%.**  **Describing serum FTA-ABS in otosyphilis (not CSF FTA-ABS)**  Not that useful given dx criteria which basically but no other data specifically in otosyphilis | *NS |
| **Jonckheere (2015)**  European Journal of ClinicalMicrobiology & Infectious Diseases **34**(10): 2041-2048. | Cross sectional, multiple univ, Belgium, mixed risk (see below)  7 assays performed: Bioplex 2200 IgG included, all others not FDA approved  Low risk patients 15.6%  Asymptomatic, at risk 61%  Symptoms of syphilis 7.5%  Equivocal results considered neg  **Gold standard:**  **True pos: TPPA pos**  **True neg; TPPA neg** | N=177, n=5 equivocal  Sensitivity 94.2 (87.8-97.8)  Specificity 59.5% (47.4-70.7) | Not stratified by stage. **Single lab test as the gold standard** | *  Bioplex IgG specificity poor using TP-PA as gold standard |
| **Jost H (2013)** BMJ Open **3**(9): e003347. | Cross sectional, serum bank specimens Georgia PH lab  FTA, LIAISON, trepsure, Captia IgG (others not FDA approved)  **Gold standard:**  **True pos: TPPA pos**  **True neg; TPPA neg** | N=290  N=109 TPPA+  N=181 TPPA-  Pos agreement: FTA-ABS 94.4%  LIAISON (100%)  Trep Sure (100%)  Captia (100%)  Neg Agreement: FTA 100%  LIAISON (99.4%)  TrepSure (98.9%)  Captia (97.2%) | Head to head agreement of multiple FDA approved assays  Not stratified by stage. **Single lab test as the gold standard** | *  Perfect positive agreement between IAs,  Captia, Liaison, Trepsure Using TPPA as gold standard  FTA had perfect neg agreement w TPPA |
| Juarez-Figueroa, L., et al. (2007). Diagnostic Microbiology & Infectious Disease **59**(2): 123-126. | Cross sectional, PH lab, Mexico  Female sex workers w/ and w/o syphilis (Chiapas)  FSW screening (Mexico City)  Prenatal screening  Determine (rapid, not FDA) and TPPA  **Gold standard:**  **True pos: VDRL/FTA-ABS+**  **True neg: VDRL/FTA-ABS+** | Group composition (% prevalence)  Group 1 FSW: 58 seropositive, 98 seronegative (38.7)  Group 2: 198 FSW (15.7)  Group 3: 200 prenatal pts (1.5)  Sensitivity/Specificity/PPV/NPV  Group 1: 98.3/95.3/93/98.9  Group 2: 88.6/100/100/97.9  Group 3: 100/100/100/100 | **Laboratory gold standard,**  High and low seroprevalence  Group 1 does not reflect true population prevalence b/c + serology oversampled | *  TPPA had sensitivity/specificity >95% except in FSW screening group, but numbers were small (N=31) |
| Kinnunen, E (1986)  Journal of the Neurological Sciences **75**(2): 205-211. | Retrospective, university hospital, Finland  Pts admitted to neurology service who received LP.  **True pos: known prior history of syphilis (with or without treatment) with current neurologic symptoms or serum VDRL and/or MHA-TP positive with current neurologic syxs**  **True neg: n/a** | N=21 pts with neurosyphilis  MHA-TP sensitivity  14/21 (67%) | **Clinically characterized cases, but**  **misclassification possible as patient could have had prior syphilis and current unrelated neuro symptoms yet classified as NS.**  **Did not require any consistent uniform lab or clinical criteria for diagnosis.** | *NS |
| Loeffelholz, M. J., et al. (2011). Clinical & Vaccine Immunology: CVI **18**(11): 2005-2006. | Retrospective, University hospital, US, incarcerated, women seen in obgyn  Bioplex IgG, RPR, TPPA  Bioplex test performance based on AI (automated index) cutoffs of 6 vs 8, stratified by population and RPR titer  Sensitivity, specificity  **Gold standard:**  True pos: **+ TPPA**  True neg: - TPPA | RPR NR: n=82 incarcerated, n=44 women at obgyn clinics  RPR ≥1:2 n-110 incarcerated, n=31 women at obgyn clinics  Sensitivity/Specificity AI6  RPR neg 52.4-57.9/92-96%  RPR ≥1:2 96.8-97.2%/100%    Sensitivity/specificity AI=8  Rpr neg: 50.9-50.4/96-100%  RPR ≥1:2 94.5-96.8/100%    (even at index value of 8, incarcerated specificity <100), but 100% for AI 8 in women with NR RPR or RPR 1:1) | **Background for analysis of index values**  RPR 1:1 not shown due to space limitations and small numbers    **Single lab test as gold standard**  **Not stratified by stage** | *  Demonstrates utility of using S/CO results to predict TP-PA positivity increased specificity if using AI of 8  Excluding women at delivery because all sample sizes are 5-7 women only |
| Malm, K., et al. (2015). Transfusion Medicine **25**(2): 101-105. | University hospital, Sweden  Cross sectional (first time blood donors, repeat donors)  LIAISON XL CIA  Sensitivity, specificity  **Gold standard : Abbot Architect results** | N=55 Architect pos  353 Architect neg  Sensitivity 100%  Specificity 100% (99.7-100) (overall) | **Single lab test as gold standard** | *  Perfect sensitivity/specificity of LIAISON with single CMIA as gold standard |
| Marangoni, A., et al. (1999).  Zentralblatt fur Bakteriologie **289**(2): 125-133. | Prospective, Univ derm clinic, Italy.  Patients with early syphilis  Blood donor samples (30)  B. burdorferi and Leptospira samples (10)  VDRL, TPHA, and FTA-ABS  Gold standard: clnical diagnosis  True pos:  Primary: chancre  Secondary: mucocutaneous lesions (Norris/Larsen)  True neg: not described, blood donors assumed to be negative | N=21 early syphilis cases (35 sera samples)  N=30 normal donors  FTA-ABS  Sens/Specifiicty  88.5%/98% | **Clinically characterized, not stratified by stage (early syphilis combined)**  **Some sera were tested twice. Unclear rationale** | ** |
| Park, B. G., et al. (2016).  Journal of Clinical Microbiology **54**(1): 163-167. | University hospital, Korea  Cross sectional, general PE and pts with suspected syphilis  Sensitivity, specificity, % agreement, kappa  Architect, Cobas, ADVIA, Sysmex, A and T, Seskisui  **Gold standard: FTA-ABS** | n=615  105 well-visits, 179 preop, 329 suspected current or known prior syphilis  Sensitivity: Architect 96.8%  Cobas 99.4%, ADVIA 99.4%  Specificity: 100% for all | Clinical data used for specimen selection but not to determine true positive/negatives status  **Single lab test as gold standard** | *  Test performance of Architect, Cobas, ADVIA,  Almost perfect sensitivity for Cobas, ADVIA, perfect specificity for all |
| Park, Y., et al. (2011).  American Journal of Clinical Pathology **136**(5): 705-710. | 2 university hospitals, Korea  Cross sectional, general medicine and derm pts.  Separate sample of pts with ANA 1:30 to 1:160  All pts tested with FTA-ABS, and Architect  400 were also tested with VDRL (chart review for discrepants between 3 tests)  Architect syphilis vs FTA or VDRL FTA ABS vs Architect  % concordance and Kappa calculated  **Gold standard: FTA-ABS (for architect performance)**  **Or VDRL result (for FTA-ABS performance)** | N=616 specimens  %Concordance:  Architect TP and FTA,99%, K 0.981 (0.965-0.996) p<0.001  N=400 specimens tested w VDRL  FTA-ABS and VDRL 85%, k 0.7 (0.631-0.739) p<0.001  Architect & VDRL, 83.8%, k 0.675  N=108 pts with + ANA  0 reactive Architect | **Single lab test as gold standard**  Clinically characterization limited to patients with discordant results between the 3 tests  unclear how they are divided. | *  nearly perfect concordance between Architect and FTA-aBS, Architect did not give FP in pts with + ANA |
|  |  |  |  |  |
| Sonmez C. Journal of Immunological Methods (2018), doi:10.1016/j.jim.2018.08.007 | **Cross sectional, PH laboratory, Turkey**  9 treponemal tests (FDA approved tests included: Architect Syphilis TP, ADVIA Centaur Syphilis, TP-HA)  **N=363 screening specimens**  **Gold standard: FTA-ABS**  **True positive: FTA-ABS reactive**  **True negative: FTA-ABS negative** | Architect  Sensitivity: 92.3% 251/272  Specificity 94.5%, 89/91  ADVIA  Sensitivity: 87.5%, 238/272  Specificity: 89.0%, 81/91 | Single laboratory test (FTA-ABS) as the gold standard, no clinical data | *  Little data on the Centaur outside of 510K so useful from that standpoint |
| Veldkamp, J. and A. M. Visser (1975). British Journal of Venereal Diseases **51**(4): 227-231. | Retrospective, national public health lab, Netherlands  “problem sera” referred to laboratory  Tested with VDRL, Kolmer, RPCF, **FTA-Abs,** TPI, ELISA (not currently in use)  **Gold standard:**  **True pos: ?unclear how characterized but staging presented**  **True neg: pts with presumed BFP but unclear why and how characterized**  ***Weakly reactive considered pos** | N=(see below for N by stage)  Sensitivity (treated-untreated, if listed)  Primary( 25/32) 96%/100%  Secondary (18/16) 100%  Latent (58) 98.2%  CV (8) 100%  NS (20) 100%  CS (10) 90%  Other (58)98.2%    N=42 pts with BFP  FTA-Abs specificity 100% | ? how clinically characterized, stratified by stage, unable to gauge how accurate gold standard is for either pos or negatives  EL/LL combined | *  Not that useful given many other studies with FTA-Abs performance stratified by stage with better characterization of the gold standard. |
|  |  |  |  |  |
| Wong, E. H., et al. (2011).  Sexually Transmitted Diseases **38**(6): 528-532. | STD clinic, prospectively collected remnant sera.  TrepSure EIA (IgG, IgM)  Tested with VDRL, TS EIA, TPPA  IgG and IgM WB used to resolve discordants, TrepID also used for EIA+, VDRL NR, TPPA NR  True positive: 1)VDRL+, TS-EIA+ and TP-PA+ or WB+  2)VDRL+ EIA-, TPPA +  3) VDRL-, EIA+, TPPA+  True neg: 1) VDRL-, EIA-, TPPA-  2) VDRL+, EIA-, TPPA-  **Gold standard**: Lab findings only using combination VDRL, TPPA and WB | N=674 specimens.  sensitivity TS EIA 279/285  97.9% (calc from figure)  Specificity: 99.1% (reported)  Microbiologist time 80 specimens  VDRL: 150 minutes  TS EIA: 120 minutes (microbiologist free time 90min, 30 min hands on)  6 False neg EIA all IgM WB+  S/CO values associated with TP-PA positivity (>8.00=99.6% TP-PA positivity) | Characterizes false negatives with WB, and false pos EIA with TrepID  Not stratified by stage  **Lab Gold standard only** | *  Strong performance of TS EIA in high prev population  Describes time differences in performance of VDRL vs EIA. |
| Woznicova, V. and Z. Valisova (2007). Journal of Clinical Microbiology **45**(6): 1794-1797. | Prospective, STD clinic, Czech R  Syxs pts, previously tx syphilis, contacts, other STDs, other pts at risk  Tested with both Captia and TPHA, if discordant, FTA-ABS, WB, and chart review, plus retesting at 3 wks and 3 months  **Gold standard: TPHA results,** plus chart review and retesting of discordants | N=1771 (1309+, 462-)  Captia syphilis G  Sens/Spec/PPV/NPV (initial)  97.7%/94.2%/97.9%/93.5%  Sens/Spec/PPV/NPV (after resolving discordants)  99%/98%, 99.3%, 97.2% | **Lab gold standard, but strengthened by chart review and repeat testing of discordants**  **Not stratified by stage** | *  Captia syphilis G high sensitivity/specificity using lab gold standard after resolution of discordants |
| Yoshioka, N., et al. (2007). Clinical Laboratory **53**(9-12): 597-603. | Cross sectional, univ hospital, Japan  Outpatients/inpatients  Tested with Architect  **Gold standard**  **True pos: RPR+ TPPA+**  **True neg: RPR and TPPA neg** | N=500 true neg  N=121 true pos  Sens/spec  100%/100% | **Lab gold standard**  **Not stratified by stage** | *  Architect 100% sens/spec using lab gold standard |
| Young, H., et al. (1989). Genitourinary Medicine **65**(2): 72-78. | Prospective, University clinic, UK  Unselected screening specimens (1280)  GUM clinic 762, prenatal 302, transfusion service (142), GP office (72)  Additional “treponemal samples” (32) and “nontreponemal sample” (9) (origins unclear) also tested  Tested with VDRL, TPHA, and Captia IgG  If VDRL+ or TPHA+, then FTA-Abs  **True pos:** TPHA and FTA-Abs positive  True neg: TPHA and FTA-Abs neg | N=1321 total specimens  N=1260 true neg  N=61 true positive  Captia IgG  Sensitivity 98.4%  Specificity 99.3% | **Lab gold standard (2 tests)**  N=37 pts with syphilis were able to be staged, but results not stratified by stage  Categorization of 32 true pos specimens unclear (? Serum bank?) | * |
| Background Only, Not Rated | | | | |
| **Citation** | **Description of study type, design, population, and setting (gold standard)** | **Reported findings, quantitative results related to key question** | **Overall quality; strengths/weaknesses or limitations** | **Relevance to the key question and/or overall importance** |
|  |  |  |  |  |
| Burns, R. E. (1975). "Spontaneous reversion of FTA-ABS test reactions." JAMA 234(6): 617-618. | Observational study of 56 patients with negative VDRL and positive FTA-ABS results. Patients were followed for 3 weeks to 21 months. | Patients without signs and symptoms of syphilis or history of dx but with negative VDRL and weakly positive FTA-ABS should be monitored for a year before a syphilis diagnosis is made.  20/56 patients with weak FTA-ABS results reverted to negative from 1 week to 8 months. | **Background only,** seroreversion of FTA-ABS weakly reactive results over time.  small sample size (56) and unsure about generalization to rest of population. No explanation of reversion. Conclusions are outdated as better diagnostics have become available and waiting for a year before diagnosing is not realistic. | Provides insight on weakly reactive FTA-ABS results and the need to monitoring these types of results before making a syphilis diagnosis. |
| Centers for Disease, C. and Prevention (2011).  - Morbidity & Mortality Weekly Report **60**(5): 133-137. | Retrospective, 4 labs, KPSC, KPNC, NYC, Chicago, US  KP-mixed screening population  NYC/Chicago-PH lab  TrepChek (KPSC, NYC)  LIAISON (KPNC)  Trepsure (KPNC, Chicago)  **Gold standard: N/A** | N=140,176 specimens total  Low prev settings (2.3% EIA+)  High prev settings (14.5% EIA+)  % non-reactive TPPA or FTA ABS, overall 31.6%  KPSC Trepcheck 60%  KPNC LIAISON 30%  KPSC TrepSure 25.2%  NYC TrepChek 12.2%)  Chicago TrepSure 18.6% | **Not test performance (background only)**  Large analysis of reverse sequence screening | Background on use of Reverse sequence algorithm and high false pos EIA in low prev populations, particularly with TrepChek  Inc. TrepSure and LIAISON |
| Centers for Disease, C. and Prevention (2008). MMWR - Morbidity & Mortality Weekly Report **57**(32): 872 875. | Retrospective, NYC ?PH labs,  Convenience sample of screening population  **Gold standard: n/a** | N=116,822 specimens  N=6587 (6%) EIA+  3664 (56%) RPR neg  433 (17%) TPPA neg  ~3% additional reactive specimens that need further testing/f/up compared to RPR based algorithm | **Not test performance**  **Descriptive data only**  No clinical interpretation of results available. | Descriptive data on early use of RSS |
| Erbelding, E. J., et al. (1997). Journal of Infectious Diseases **176**(5): 1397-1400. | Prospective, multi-university, US  IDUs, q 6 month HIV and syphilis testing. (total n=1117)  RPR and FTA-ABS performed  If RPR>1:8 yet FTA-ABS-, then immunoblot performed on selected BFP with titer ?1:8 (selection criteria not described)  Gold standard: N/A | N=112 BFP reactors  1) 68 (61%) were chronic BFP  **2) 25 (31%) RPR>1:8 at some point**  3) 5 (4.5%) converted to FTA-ABS+  4) 4 (3.6%) documented FTA-ABS+ in past | **Descriptive data only on false negative FTA-ABS in HIV+ IDU with RPR titers of 1:8 or greater during follow-up.**  **Only 5 patients selected among 25, and selection criteria unclear. 3/5 demonstrated +17F and 47F antigens** | Descriptive data, False negative FTA-aBS can occur among pts with HIV |
| Haas, J. S., et al. (1990).  Journal of Infectious Diseases **162**(4): 862-866. | Retrospective, university hospital serum banks, CA, US  MSM participating in 2 cohort studies of HIV (both pos /neg) all had syphilis serology, HIV and Tcell counts.  VDRL, MHA-TP or FTA-ABS performed, must be trep test pos for inclusion  Gold standard: N/A | N=109 included  19 HIV- MSM  80 HIV+ MSM  No seroreversions among HIV-  13/80 seroreverted (16.3%)  Significant predictors for loss of trep test postivitiy | **Not test performance, background data only** | Loss of trep test positivity in HIV related to first vs repeat syphilis, lower VDRL, more advanced HIV/AIDS |
| Henrich, T. J. and S. Yawetz (2011). Sexually Transmitted Diseases **38**(12): 1126-1130. | Cross sectional, university hospital US, Captia IgG  Screening population at univ hospital. Descriptive data on IgG EIA positivity, % concordance with TPPA  Gold standard N/A | N=34,251 samples, 1.8% positivity  79% confirmed with TPPA  Lower confirmation if age <40, OB/GYN service patient, or female | **Not test performance (background only)**  describes % confirmation of Captia EIA in low prevalence population | Background on Captia demonstrates more FP in lower prevalence populations |
| Janier, M., et al. (1999)  Dermatology **198**(4): 362-369 | Prospective, university hospital, France  MSM, HIV+, non IDU, all w/ treated syphilis, positive baseline treponemal test, followed for 3 follow up visits (q 6 months)  Control group; 49 HIV neg MSM, similar inclusion criteria  Survival analysis performed to examine seroreversion of FTA-Abs y HIV status  Gold standard: N/a, descriptive | N=69 in study group  N=49 controls  Seroreversion more frequent in HIV + patients (log rank p=0.001) (still significant after adjustment for stage, time since last episode of disease, and age)  Loss of FTA-ABS positivity related to low CD4+ count at baseline (CDC stage 3) (p-0.003)  HIV-negative: No time effect for FTA-ABS seroreversion | **Descriptive data only, seroreversion not related to stage at time of treatment** | Descriptive data, loss of FTA-ABS positivity related to low CD4 count |
| Johnson, P. D., et al. (1991). AIDS **5**(4): 419-423 | Case control, Public hospital, Aust  Paired samples from pts (spanning 3 y) with AIDS/syphilis and HIV-controls/ (random sampling of serum excluding samples from yaws-endemic areas or MSM clinics)  Tested with TPHA, FTA-ABS (controls also tested w HIV)  Fall in Trep Ab (reduction in TPHA titer 4 fold, or 2+ reduction in FTA-ABS)  **Gold standard: n/A descriptive** | N=29 patients with paired specimens  N=29 controls  12/29, 41% of AIDS patients showed fall in either TPHA or FTA-ABS antibody.  4/29 (14%) of controls had fall in antibody  P=0.02 | **Descriptive data only, no information on stage at time of treatment** | Descriptive data, Loss of trep-specific Ab can occur among pts with HIV more commonly than among HIV neg controls |
|  |  |  |  |  |
| Park IU (2011)  Journal of Infectious Diseases **204**(9): 1297-1304. | Managed care organization, US  Cross sectional, mixed risk screening population  Descriptive data on testing and differences among discordant pts (CIA+, RPR-) according to TP-PA status.  LIAISON CIA index values correlated to TP-PA  Gold Standard: N/A | N=255 CIA+, RPR-  TPPA pos (184), TPPA neg (71)  87% of TPPA pos who were retested stayed same.  7/31 (23%) isolated CIA+ seroreverted to CIA-  1 isolated CIA+ seroconverted to RPR pos in 6 months  LIAISON S/co of 12.00 associated with 100% TP-PA positivity | Background only  Not all patients retested  S/co cutoff data useful  Descriptive data, no test performance | Descriptive data on sero-reversion of isolated CIA+  S/co cutoff 12.00 associated with 100% positivity |
| Romanowski, B., et al. (1991). " Annals of Internal Medicine **114**(12): 1005-1009. | Retrospective, PH lab, Alb, CAN  All cases of syphilis dx 1981-1987  Tested with RPR and MHATP and/or FTA ABS, plus clinical criteria  Examined for seroreversion at 3, 6, 12, 24, 36 months  Excluded: pregnant, negative serology before tx, treatment failure, loss to f/up, clinical relapses  **Gold standard true pos:**  **Primary: + DF, or chancre plus seroconversion**  **Secondary: typical rash, mucous membrane lesions, Condyloma lata—DF pos**  **EL: asymptomatic, contact to P/S in last year or untreated P/S in last year**  ?patients tested with more than 1 trep assay? | N=882 evaluable pts  N=857 primary  N=182 secondary  N=50 EL  *All pts with seroreversion were experiencing 1^st^ episode of primary syphilis  N=616 patients w/ 1^st^ episode primary, MHA-TP+  N=259 patients w/ 1^st^ episode primary, FTA-ABS+  Seroreversion increases with time, by 36 months, 13% (SE 2%) were MHA-TP neg (out of 126 pts)  23.8% were FTA-ABS neg (out of 55 pts) | **Descriptive data**  **Large sample size, seroreversion analysis had large loss to f/up (retrospective so to be expected)**  **Not stratified by HIV status b/c in 1985 seroprevalence of HIV was still low** | 24% of pts seroreverted FTA-aBS after tx for primary syphilis in cohort w low HIV prevalence |
| Ross, J., et al. (1991). Genitourinary Medicine **67**(5): 408-410 | Retrospective, GUM clinic, UK  **False pos**: EIA index >0.9  VDRL, TPHA, FTA-ABS and IgM EIA negative, no clinical signs or symptoms | N=12842 GUM patients screened.  197 (1.5%) False pos (not different according to STD)  N=10314 prenatal  82 (0..8%) false pos (p< 0.01) compared to GUM | **Descriptive data**  **FP characterized by both clinical and extensive lab criteria** | Isolated EIA pos more common in GUM-STD patients than antenatal testing |
| Sun, R., et al. (2013).  Chinese Medical Journal **126**(2): 206-210. | University hospital, cross sectional  Inpatients with syphilis (old or new)  TP-PA and RPR pos  TP-PA and RPR neg but contact to syphilis or “typical” presentation  **Gold standard: N/a** | N=69  N=17 primary  N=14 secondary  N=11 teritary  TP45 IgM was most frequent in pts with primary disease (64.7%)  TP15 igM only detected in those with tertiary syphilis  Tx reduced Tp17 IgG and TP47 IgG | **Not test performance, background only**  Small numbers in each stage  Descriptive data only, no test performance by stage | Descriptive background on TP antigens and their appearance |
